# Delivery of Oligonucleotide Therapeutics: Chemical Modifications, Lipid Nanoparticles, and Extracellular Vesicles

**DOI:** 10.1021/acsnano.1c05099

**Published:** 2021-09-10

**Authors:** Jeremy P. Bost, Hanna Barriga, Margaret N. Holme, Audrey Gallud, Marco Maugeri, Dhanu Gupta, Taavi Lehto, Hadi Valadi, Elin K. Esbjörner, Molly M. Stevens, Samir El-Andaloussi

**Affiliations:** † Department of Laboratory Medicine, Karolinska Institutet, Huddinge 14152, Sweden; ‡ Department of Medical Biochemistry and Biophysics, Karolinska Institutet, Stockholm 17177, Sweden; § Department of Biology and Biological Engineering, Chalmers University of Technology, Gothenburg 41296, Sweden; ∥ Advanced Drug Delivery, Pharmaceutical Sciences, R&D, AstraZeneca, Gothenburg 43150, Sweden; ⊥ Department of Rheumatology and Inflammation Research, Institute of Medicine, Sahlgrenska Academy, University of Gothenburg, Gothenburg 41390, Sweden; # Institute of Technology, University of Tartu, Nooruse 1, Tartu 50411, Estonia; ¶ Department of Materials, Department of Bioengineering, Institute of Biomedical Engineering, Imperial College London, London SW7 2BU, United Kingdom

**Keywords:** oligonucleotide, oligonucleotide delivery, intracellular trafficking, endosomal escape, RNA therapeutics, lipid nanoparticles, extracellular vesicles, cellular uptake

## Abstract

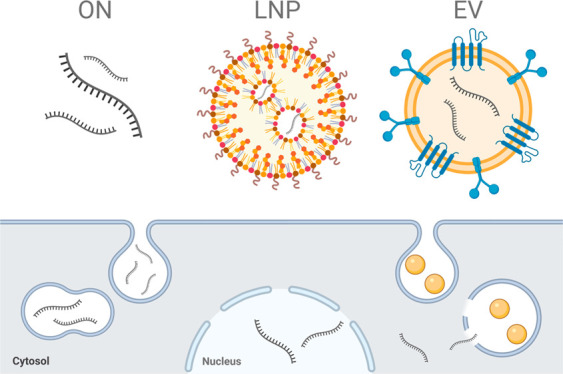

Oligonucleotides (ONs) comprise a rapidly growing class of therapeutics. In recent years, the list of FDA-approved ON therapies has rapidly expanded. ONs are small (15–30 bp) nucleotide-based therapeutics which are capable of targeting DNA and RNA as well as other biomolecules. ONs can be subdivided into several classes based on their chemical modifications and on the mechanisms of their target interactions. Historically, the largest hindrance to the widespread usage of ON therapeutics has been their inability to effectively internalize into cells and escape from endosomes to reach their molecular targets in the cytosol or nucleus. While cell uptake has been improved, “endosomal escape” remains a significant problem. There are a range of approaches to overcome this, and in this review, we focus on three: altering the chemical structure of the ONs, formulating synthetic, lipid-based nanoparticles to encapsulate the ONs, or biologically loading the ONs into extracellular vesicles. This review provides a background to the design and mode of action of existing FDA-approved ONs. It presents the most common ON classifications and chemical modifications from a fundamental scientific perspective and provides a roadmap of the cellular uptake pathways by which ONs are trafficked. Finally, this review delves into each of the above-mentioned approaches to ON delivery, highlighting the scientific principles behind each and covering recent advances.

## RNA Therapeutics Overview

The field of nucleic-acid-based therapeutics is entering an era highlighted by increased clinical success and intense interest by pharmaceutical and biotech industries. The continuous improvements in nucleic-acid-based drug compositions along with the extensive mapping of genetic targets are fueling the exponential growth of applications for these therapies.

The Food and Drug Administration (FDA) broadly defines gene therapy as therapy that seeks to modify or manipulate the expression of a gene or to alter the biological properties of living cells for therapeutic use.^1^ This is often accomplished by delivering exogenous nucleic acid sequences to the cells of interest. These sequences may include DNA, RNA, a range of synthetic analogues which are based off of these two natural nucleic acids, or quite often a mix thereof. In addition, functional delivery is often boosted by addition of other bioactive compounds and macromolecules such as lipids and peptides. The ability to construct these complex molecules has resulted in a wave of medical innovation. Using the approaches outlined in this review, nucleic-acid-based therapies can be used to treat a range of diseases which have remained unreachable through classical pharmacological intervention.

Recently, the outbreak of the SARS-CoV-2 virus has prompted a significant shift of focus into the RNA therapeutics space as Moderna Therapeutics and Pfizer/BioNTech developed mRNA vaccines to combat the ongoing pandemic. As much of the media focus highlighted the novelty of using RNA in a vaccine, it could be overlooked that an equally important feat was the development of the lipid nanoparticle (LNP) formulations that enabled cellular administration and hence therapeutic functionality of the mRNA. The development of these vaccines was also dependent on the ability to mass-produce RNA therapeutics. Broadly, RNA therapeutics can be classified as either mRNA-based or small RNA-based. Both classes are growing rapidly in scientific and therapeutic interest.

Oligonucleotides (ONs) are small nucleic acid strands which are typically 15–30 base pairs in length and contain various chemical modifications to favorably alter their behavior. These short sequences can bind with exceptional specificity and affinity to almost any RNA sequence, whether in pre-mRNA, mRNA, ribonuclearproteins, or miRNAs. ONs can furthermore be designed to assemble to a specific 3D conformation capable of binding proteins. The inherent combinatorial nature of nucleic acid sequences provides an immediate advantage in terms of drug design; any nucleic acid target can be addressed, while the pharmacokinetic properties of the drug can be tuned separately. The pharmacokinetics of an ON are generally determined by the backbone chemistry, while the target is determined by the nucleotide sequence. In contrast, small-molecule compounds are often extremely limited in their ability to separate these two characteristics.^2^

Still, significant hurdles remain for widespread use of ONs and other nucleic-acid-based therapeutics, which must be overcome at almost all levels from drug design to functional delivery:

- Chemical: the therapeutic molecules must have an adequate half-life and stability.
- Cellular: the molecules must be able to enter cells in adequate concentrations and usually cross biological lipid membranes to reach their sites of action. Additionally, the ONs should be able to effectively target specific cells and evade others.
- Immunological: the molecules should stimulate appropriate immunorecognition outcomes but should not induce an undesired immune response.
- Tissue: the targeted cells must have been corrected in a high enough quantity and in a time-dependent manner to overcome the tissue’s weakness or defect.
- Clinical (patient-facing): the specificity of the drug to the desired tissues must be high and toxicity must be low, and off target affects must be minimal.
- Clinically (population-facing): the drug must be readily scalable and affordably produced to be a practical therapeutic, with a predictable behavior across the population.

These barriers have been known to researchers for years; however, there are still improvements which need to be made. In particular, the infamous “endosomal escape” problem has proven difficult to solve. This involves the inability of biomolecules such as ONs to permeate endosomal membranes and gain access to the cytosol. Three promising approaches to overcome this barrier have emerged: chemically altering naked ONs to give them favorable properties, formulating synthetic lipid-based nanoparticles capable of inducing endosomal release, and loading ONs into biological vesicles to exploit natural delivery pathways. The aim of this review is to summarize the recent clinical advancements of ON therapeutics and to discuss in-depth the underlying scientific developments regarding the chemistry and uptake of ONs, specifically through three delivery strategies: administering chemically modified ONs, formulating lipid nanoparticles to deliver ONs, and designing extracellular vesicles to deliver ONs.

### Commercial Advancements in ON Therapeutics

Although ONs were shown to target RNA and inhibit protein translation in 1978,^3^ it took 20 years before patients received a commercial ON treatment. ONs do not fall within the scope of advanced therapy medicinal product (ATMP) regulations as they are classified as chemical drugs by the FDA and the European medicines agency (EMA).^4^ Ionis Pharmaceuticals (formerly Isis Pharmaceuticals) earned FDA approval for an ON drug in 1998 with the development of Vitravene (fomivirsen), which was used for the treatment of cytomegalovirus (CMV) retinitis in AIDS patients.^5^ The problem of tissue targeting was overcome by administering the drug *via* intraocular injection. However, the commercialization of this ON was not entirely successful. Fomiversen’s market share has fallen considerably due to the introduction of a small-molecule drug for the same condition. Additionally, the next two ONs to receive FDA approval, Macugen (pegaptanib) and Kynamro (mipomersen), experienced difficulties after they made it to market.^6^ Both ONs failed to maintain strong market share due to competing antibody-based and small-molecule therapeutics. However, 2016 marked a turning point in two significant FDA approvals: Exondys 51 (Eteplirsen) for Duchenne muscular dystrophy (DMD)^7^ and Spinraza (nusinersen) for spinal muscular atrophy (SMA), discussed further below.^8^ The complete list of FDA-approved ON therapies as of today is shown in Table 1.

**Table 1:** Current FDA-Approved ON Therapeutics^a^

drug name	developer	FDA approval	indication	target	class, Mer^b^	chemical modifications
Vomivirsen (Vitravene)	Ionis Pharm. and Novartis Opthalmics	Aug 26, 1998	CMV retinitis	viral IE2 mRNA	DNA, 21	•PS backbone
						•5′ CpG motif
Macugen (Pegaptanib)	NeXstar	Dec 14, 2004	retinal AMD	VEGF-165	aptamer, 27	•PS 3′-3′ deoxythymidine cap
						•2′-OMe purine ribose sugars
						•2′-F pyrimidine ribose sugars
						•PEG conjugation
Kynamro (Mipomersen)	Ionis Pharm. and Genzyme	Jan 13, 2013^b^	HoFH	apoB mRNA	gapmer, 20	•PS backbone
						•2′-O-MOE 5-mer regions
Exondys 51 (Eteplirsen)	Sarepta Therapeutics	Sep 19, 2016	DMD	dystrophin pre-mRNA	SSO, 30	•PMO
Defitelio (Defibrotide)	Jazz Pharmaceuticals	Apr 1, 2016	sVOD	nonspecific^c^	mixed (avg. 50)	•PO backbone
						•single and double stranded
Spinraza (Nusinersen)	Ionis Pharm.	Dec 23, 2016	SMA	SMN1 and SMN2 pre-mRNA	SSO, 18	•PS backbone
						•2′-O-MOE
						•5-methyl-C
Onpattro (Patisiran)	Alnylam	Aug 8, 2018	hATTR	TTR mRNA	siRNA, 19 pass. and 21 guide	•double stranded
						•2′-OMe uridines
						•LNP encapsulation
Tegsedi (Inotersen)	Ionis Pharm. and Akcea Therapeutics	Oct 5, 2018	hATTR	TTR mRNA	gapmer, 20	•2′-O-MOE
Givlaari (Givosiran)	Alnylam	Nov 20, 2019	AHP	ALAS1 mRNA	siRNA, 21 pass. and 23 guide	•double stranded
						•partial 2′-F
						•partial 2′-OMe
						•partial PS backbones
						•GalNAc conjugation
Golodirsen (Vyvondys 53)	Sarepta Therapeutics	Dec 12, 2019	DMD	dystrophin pre-mRNA	SSO, 25	•PMO
Viltepso (Viltolarsen)	NS Pharma	Aug 12, 2020	DMD	dystrophin pre-mRNA	SSO, 21	•PMO
Oxlumo (Lumasiran)	Alnylam	Nov 23, 2020	PH1	HAO1 mRNA	siRNA, 21 pass. and 23 guide	•double stranded
						•partial 2′-F
						•partial 2′-OMe
						•partial PS backbones
						•GalNAc conjugation
Amondys 45 (Casimersen)	Sarepta Therapeutics	Feb 25, 2021	DMD	dystrophin pre-mRNA	SSO, 22	•PMO

a Abbreviations: CMV, cytomegalovirus; PS, phosphorothioate; AMD, age-related macular degeneration; 2′-OMe, 2′-O-methyl; 2′-F, 2′-fluoro; PEG, polyethylene glycol; HoFH, homozygous familial hypercholesterolemia; 2′-O-Moe, 2′-O-methoxyehtyl; DMD, Duchenne muscular dystrophy; PMO, phosphorodiamidate morpholino oligomer; sVOD, severe hepatic veno-occlussive disease; PO, phosphodiester; SMA, spinal muscular atrophy; hATTR, hereditary transythyretin amyloidosis; LNP, lipid nanoparticle; AHP, acute hepatic porphyria; GalNAc, *N*-acetylgalactosamine; PH1, primary oxaluria type 1; FGF2, fibroblast growth factor 2.

b European Medicines Agency refused marketing authorization on Dec 13, 2012.

c It is hypothesized that the DNA oligomers mimic heparin, binding proteins, primarily FGF2.

ONs which have not met their clinical trial end points also continue to provide valuable insights into the development of future drugs. For example, in 2016, Alnylam Pharmaceuticals had two ON therapies (siRNA-based) in phase III trials for human transthyretin amyloidosis (hATTR), patisiran and revusiran. While both drugs utilized delivery strategies to target the liver, they differed in their delivery approach—revusiran was administered subcutaneously and was composed of siRNA conjugated to the carbohydrate *N*-acetylgalactosamine (GalNAc); patisiran was an siRNA formulated within a lipid nanoparticle (LNP). Revusiran, although capable of efficient delivery, never gained FDA approval due to a high mortality rate in a phase III study.^9^ Although the discontinuation of revusiran was a major setback to Alnylam and the ON field, the GalNAc conjugation delivery approach later reached clinical relevance when Alnylam received approval for givosiran in 2019. Givosiran targets the liver for the treatment of acute hepatic porphyria (AHP).

Since fomiversen received approval in 1998, the field of antisense technology has matured significantly, with some products advancing to approval quickly while others are hindered due to a number of scientific and regulatory factors.^10^ Additionally, disease targets have been mapped across a range of diseases, providing numerous opportunities for intervention with ONs. At the beginning of 2021, there were over 200 clinical trials registered for ONs in the oncology space in the USA.^11^ Further, current clinical trials are using ONs to treat cardiovascular diseases, metabolic diseases, and, among others, infectious diseases such as hepatitis.^12^ In order to understand where the next medical and scientific advancements lie, the underlying science of the field must be considered.

## Classes of ON Therapeutics

**Figure 1:**
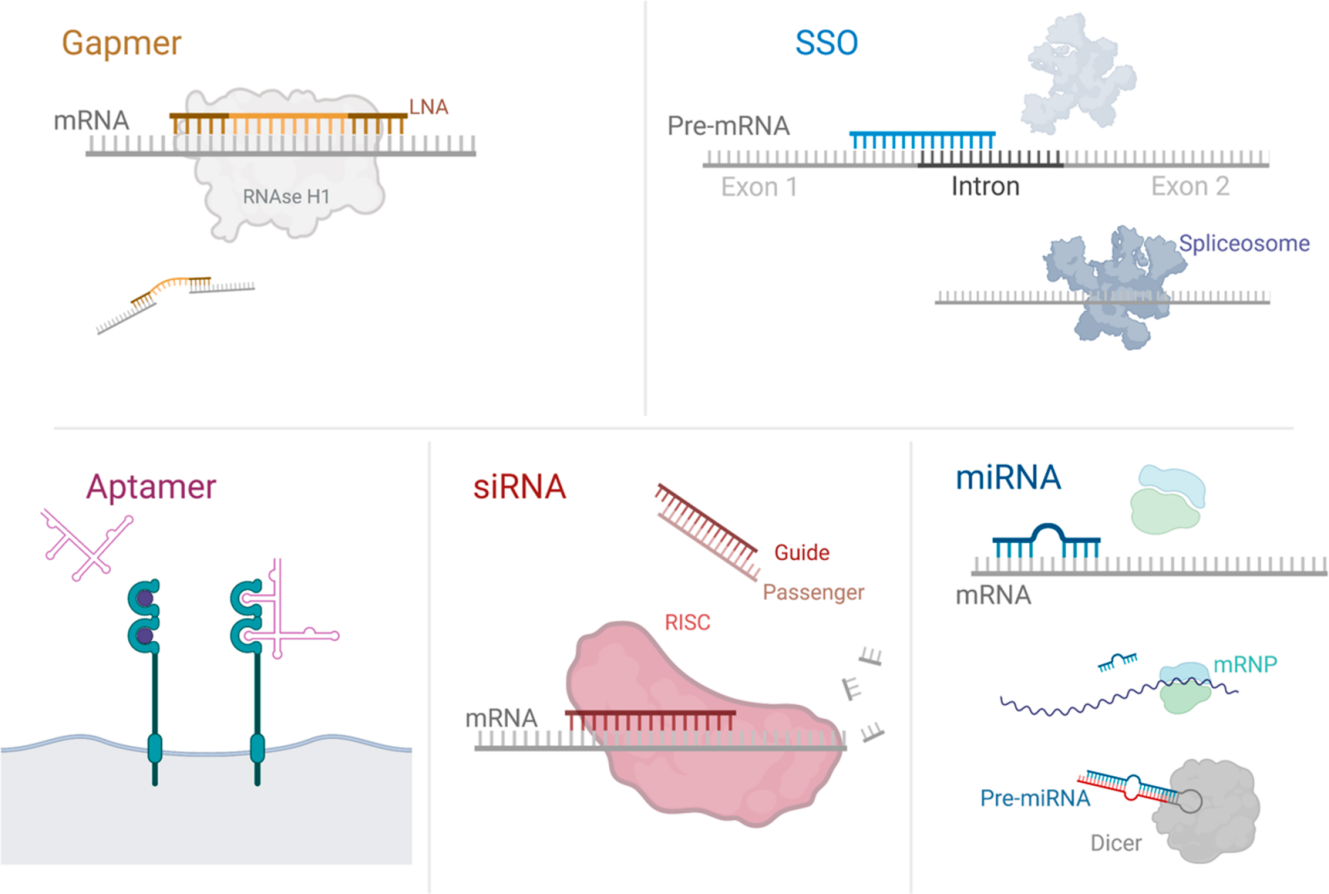
Commonly used types of ONs and their target species. Five types of ONs are discussed in this review. Gapmers target mRNA with high affinity thanks to LNA base pairs. The unmodified nucleotides in the central region allow RNase H1 to bind, degrading the mRNA. Splice-switching ONs target splice sites of pre-mRNA, preventing the splicing machinery from forming and altering the resultant mRNA. Aptamers have a 3D structure which mimics the ligands of the proteins they target with high specificity and affinity. The guide strand of double-stranded siRNA guides the RISC to the target mRNA, leading to RISC-mediated degradation. miRNA is activated by cleavage by Dicer, where it binds to mRNA preventing the formation of RNP complexes and ultimately destabilizing the mRNA. Abbreviations: SSO, splice switching ON; siRNA, short inhibiting RNA; RISC, RNA-induced-silencing complex; miRNA, micro-RNA; mRNP, mRNA–protein complex. Figure created in BioRender.

ONs have been developed to target DNA, RNA, protein regions, and even post-translational modifications. Here, the most clinically relevant classes of ONs are summarized along with examples of FDA-approved ON therapies. A general illustration of the classes discussed herein is found in Figure 1. Several other types of ONs have been identified and synthesized. A recent review by Smith and Zain comprehensively reports the broad range of ON therapeutic strategies in more depth.^13^ Additionally, other classes may emerge as we expand our understanding of the many regulatory roles of RNA.

### Gapmers

Gapmers have historically been the most widely used class of antisense ON (AON) therapeutics.^13^ The sequence of a gapmer is fully complementary to its target RNA strand so that binding occurs *via* Watson–Crick base pairing. The gapmer contains a middle region of 6–10 DNA nucleotides, which is flanked on either end by 3 to 5 modified oligonucleotides. These modified nucleotides should contain chemical modifications (discussed below) that increase both nuclease resistance and target binding affinity.^13^ The name “Gapmer” was coined for this DNA “gap” between the modified nucleotides. Gapmers operate by binding their target mRNA sequence and sequentially recruiting RNase H1, an endogenous RNase which cleaves the RNA strand of a DNA–RNA duplex in both the cytoplasm and the nucleus.^14^ Gapmers have received particular attention for their ability to successfully silence genes in cells which are traditionally difficult to transfect, such as T-cells.^15^ They have shown promise *in vivo* for their gene-silencing potency, even showing a higher potency than siRNAs in certain cases.^16^ Inotersen is an approved gapmer therapeutic which targets transthyretin (TTR) mRNA to reduce pathogenic TTR aggregation in individuals with hereditary transthyretin amyloidosis. In a phase I clinical trial, a 22 day schedule of subcutaneous administration of 300 mg of inotersen led to reductions of plasma TTR protein up to 76% for 4 weeks after the last dose,^17^ and the drug is now used in the treatment of the polyneuropathy of hereditary transthyretin-mediated amyloidosis in adults.

### Splice-Switching ONs (SSOs)

SSOs are a class of steric block ONs that emerged in the early 1990s.^18^ While gapmers lead to degradation of the mRNA, SSOs redirect splicing without depleting mRNA transcript levels, which is particularly valuable in cases of disease where abnormal splicing depletes functional protein. SSOs work by masking a splice site or silencing and enhancing elements in exons and introns, leading to the failure of the spliceosome to assemble and/or read properly. Pre-mRNAs with weak splice sites are generally better suited for targeting with SSOs than with gapmers as they are already prone to produce various protein isoforms. SSOs can work to either restore function in dysfunctional splice variants or to impede the splicing of pathological variants including viral transcipts.^19,20^

SSO structure differs from gapmer structure, as SSOs should be designed to prevent RNase recruitment as their purpose is not to induce mRNA degradation. SSOs are designed to utilize chemical modifications which increase their stability, cellular delivery, and binding affinity. These include for example morpholinos, 2′-OMe phosphorothioate, LNAs, and other modified nucleotides.^21,22^ As inferred by Table 1, SSOs have proved particularly useful for treatment of SMA and DMD. In 2009, morpholino SSOs were able to achieve a dose-dependent restoration of functional dystrophin without any adverse events reported.^22^ Since 2016, half of the FDA-approved ON therapeutics have been SSOs. Eteplirsen is a morpholino SSO which targets exon 51 of dystrophin pre-mRNA, leading to exon-skipping in the DMD gene which yields a truncated yet functional dystrophin protein. The phase II study of eteplirsen revealed that it was tolerated very well when treated up to 20 mg/kg for 12 weeks.^23^ However, the results of this study and yet another phase II trial have only been able to induce modest increases of dystrophin expression. Furthermore, the methods used in these clinical trials were heavily disputed, leading to a delay in market approval.^24,25^ Ultimately, the FDA approved eteplirsen in 2016. Another SSO with a significant history is nusinersen, a modified SSO which targets survival motor neuron 1 and 2 (SMN1 and SMN2, respectively) pre-mRNA in patients with SMA. The severity of SMA is dependent primarily on the absence of SMN1 and the absence of SMN2 to a lesser extent. The more functional transcripts of these genes a patient has, the less severe the disease outcome will be. Nusinersen works by masking a weakened splice site to restore inclusion of exon 7 into SMN2, enhancing production of the full-length protein variant. Importantly, as only a small fraction of ONs can cross the blood–brain barrier, nusinersen must be administered intrathecally by lumbar puncture. In two separate phase III studies, nusinersen was found to drastically improve motor function in young children.^26,27^ Both trials were ended early due to favorable outcomes in the drug-treated subjects. Additionally, a phase II trial in which nusinersen was given to presymptomatic children found that most children (>88%) achieved normal motor function development.^28^

### Aptamers

Aptamers are synthetic structural binding elements composed of nucleic acids.^13^ They are single stranded with a function reliant on their folded 3D structure. Aptamers are produced *in vitro* through a process called systematic evolution of ligands by exponential enrichment (SELEX), a method of ligand screening for specificity.^29,30^ Aptamers have been nicknamed “chemical antibodies” due to their ability to recognize and bind proteins in a similar fashion to protein antibodies.^31^ It was previously believed that aptamers were immunoinert; however, it has since been demonstrated that single-stranded DNA ONs have the potential to activate immune responses when administered in blood.^32^ The advantages of aptamers over antibodies are numerous: aptamers exhibit better uptake due to their relative smaller molecular weight compared to proteins and they can be chemically synthesized at scale, whereas protein antibody production is a far more laborious process.^33^ Additionally, aptamers can be rapidly “turned off” with administration of a complementary ON strand.^34,35^ Despite these numerous advantages, aptamers are not yet a competitive therapeutic alternative to protein antibodies as the only FDA-approved aptamer, pegaptanib, is losing market share to a more efficacious monoclonal antibody.^36^

### Micro-RNA (miRNA)

miRNAs are naturally present double-stranded RNA ONs, usually found within intronic regions of RNA. They undergo a two-step RNase III-dependent processing to create primary miRNA (pri-miRNA) hairpin structures which are cleaved into an active form by Dicer, an endoribonuclease enzyme.^37^ The Dicer cleavage forms a 21 nucleotide, double-stranded miRNA. miRNAs work through the RNA interference mechanism, which is the main post-transcriptional gene-silencing mechanism. This includes the miRNA binding in the 3′ untranslated region of mRNA, leading to loss of the poly-A tail and consequent mRNA destabilization.^38^ As a result, the mRNA is prevented from forming mRNA–protein structures (mRNPs) and therefore protein translation from the mRNA cannot be initiated.^39^ Additionally, it is believed that miRNAs can switch roles between translation repression and activation according to cell cycle phase.^40^

Several hundreds of endogenous miRNAs have been identified and extensively characterized. They have been investigated and mapped as biomarkers in a diagnostic approach toward cancer detection.^41^ miRNAs found in circulation may also be present as a result of their role in immune system communication. miRNAs have both physiological and pathological roles in the immune system.^42−44^ There exists an opportunity to further characterize the connection between miRNAs and the immune system, demonstrated by the fact that several clinical trials are being conducted for therapeutics which target miRNAs have led to immunological adverse effects.^45^

Therapeutic strategies involving miRNAs fall into two categories: antimiRs and miRNA mimics. AntimiRs, as their name suggests, are antagonists to miRNAs and function by binding and deactivating endogenous miRNAs.^46^ miRNA mimics, on the other hand, are exogenous and act to boost the activity of the endogenous miRNAs. Both antagomirs and mimics can be synthetically modified to increase their stability and binding affinity.

### Short Interfering RNA (siRNA)

siRNAs are usually 20–25 base pairs long and delivered to the cell as a double-stranded RNA duplex, which includes a guide strand and a passenger strand. The guide strand is designed to be completely complementary to their target sequence. Once in the cytosol, the guide strand dissociates from the passenger strand and binds to endogenous Ago2, the key nuclease component of the RNA-induced-silencing complex (RISC). The guide strand then directs this protein complex to the target mRNA.^47^ While gapmers bind their target sequence and then recruit the nuclease, siRNA binds the nuclease complex (RISC) and then targets the mRNA. Due to their intrinsic ability to specifically silence gene expression by degrading mRNA, siRNAs have most commonly been utilized to downregulate protein expression levels.

Ago2 and the RISC complex have specific structural requirements that the ONs must contain in order to bind. This limits the extent of chemical modification that the siRNA can undergo and in turn its stability and cellular uptake. So far, three siRNAs which contain partially modified bases have shown clinical success and attained FDA approval, beginning with patisiran.^48^ Patisiran is a modified siRNA which is formulated with a lipid nanoparticle (LNP) carrier to target TTR mRNA. Administration of 0.3 mg/kg patisiran every 3 weeks for 18 months was able to decrease TTR levels up to 87.8%.^49^

## Chemical Modifications of RNA ONs

Unmodified DNA and RNA exhibits minimal therapeutic activity because they are rapidly degraded, exhibit poor cellular uptake and/or are filtered out of blood in biological environments. In order to overcome these problems, enormous efforts have been made to identify ON chemistries, naturally occurring and synthetic, which improve the target binding affinity, plasma stability, resistance to degradation, and pharmacokinetics. Most of the currently utilized chemistries for ONs have been resolved and characterized for several years and the fact that they are still widely used implies that they have proven to be highly effective.^50^ The structure of an ON can be modified at all three of the functional regions—the nucleobase, the carbohydrate, and the phosphodiester linkage.

### Nucleobase Modifications

Base modifications are often used when stronger Watson–Crick base pairing is needed. By modifying the base of the nucleotide, a higher affinity for the target nucleotide can be achieved. This increases the thermal stability of the duplex formed between the ON and its target RNA, which can greatly increase the activity when using ONs for mRNA-silencing. If the ON has bound its target RNA tightly enough, splice sites can be hidden, ribosomal assembly can be prevented, and translation can be inhibited.^51^ Additionally, base modifications can act to strengthen the 3D structure of aptamers.^52^ It must be noted, however, that the increased binding affinity may increase the risk of off-target binding and thus the risk of adverse effects.

The 5-position of pyrimidines is a commonly utilized location for modification.^53^ The most commonly utilized is the “5-methyl-C” chemistry in which a methyl group is attached. The increase in stability is attributed to the stacking of the methyl groups between the nucleobases in the major groove of the formed RNA duplex. Importantly, the modification seems to act unfavorably if it is too large, as another common 5-position modification, the 5-propynyl group, has been shown to impede siRNA-mediated silencing due to the fact that it is a relatively bulky modification and the RISC cannot properly attach.^54^

There exist further synthetic base modifications including tricyclo DNA (tcDNA) and others, covered more in depth in a recent review by Smith and Zain.^13^

### Carbohydrate Modifications

The deoxyribose in DNA and the ribose in RNA can be modified to increase the oligo’s stability against nuclease degradation, greatly enhancing its pharmacokinetic half-life from a matter of days to weeks.^55^ This is due to the fact that an electron-withdrawing group on the 2′ carbon of ribose can induce the ribose to pucker into a conformation which is favorable for duplex formation.^13^ This is the reason RNA–RNA duplexes are more stable than DNA–DNA duplexes, and several ON chemistries aim to replicate this structure. Hybridization analysis of several 2′-modifications revealed that not all modifications enhance RNA affinity equally.^56^ The most widely utilized modification is the 2′-O-methyl (2′-O-Me) in which a methyl group is attached. Other common modifications include 2′-O-methoxyethyl (2′-O-MOE), and 2′-fluoro RNA (2′-F-RNA).

The carbohydrate can also contain modifications in which “lock” the nucleotide into its north conformation. By bridging the 2′-O to the C4′ with a methylene linkage, a drastic increase in duplex stabilization can be achieved. This chemistry, the locked nucleic acid (LNA), has since been effectively used in siRNAs, gapmers, splice-switching ONs, and antagomirs.^57^

### Phosphodiester Linkage Modifications

The backbone of a nucleic acid strand is the repetitive sequence between sugar group and phosphodiester linkage which effectively gives the strand its helix shape. The backbone is also the target for degradative endo- and exonucleases. The nonmodified phosphodiester (PO) linkage of human DNA and RNA has several unfavorable pharmacokinetic and distribution properties for its use as a therapeutic, including a short half-life in circulation due to nuclease susceptibility and low serum protein binding ability.

The most commonly employed chemical modification in both research and clinical use is the phosphorothioate (PS) modification. In the PS backbone, a nonbridging oxygen of the phosphodiester linkage is replaced with a sulfur atom. The foremost advantage to this substitution is that the PS-ON gains resistance against nuclease degradation.^58^ As the chemistry was studied further, it was also found that the PS backbone was inducing an increase in tissue uptake.^59^ When PO-ONs are administered by injection *in vivo*, they are rapidly degraded into their constituent monomers and cleared in urine without any significant delivery to tissue.^60^ However, PS-modified ONs show much better tissue uptake with as little as 10% of the administered dose cleared in urine, and even the cleared ON had not been degraded.^61^ It is believed that PS-ONs are retained in circulation due to their increased binding affinity for serum proteins such as albumin, which helps them to evade blood clearance long enough to reach their target tissues. PS-ONs accumulate most readily into kidney and liver, with the kidney having 84:1 organ-to-blood ratio and the liver having 20:1 at 2 h after injection.^60^

Interestingly, it was later found that the PS backbone exists in nature in certain bacteria DNA.^62^ In nature, some bacteria contain CpG motifs, C-G dinucleotides which are partially modified to contain PS backbone linkages. CpG dinucleotides have been found to bind TLR9, triggering immune stimulation and B-cell activation.^63^ This finding has been the basis for many therapeutic developments in which CpG-containing ONs are used as immunostimulatory therapeutics against allergies, cancer, and a range of other immunological disorders.^64^

Importantly, the activity of PS-ONs cannot always be correlated between *in vivo* and *in vitro* experiments due to the delicate dependency on time, temperature, concentration, and cell line used.^65^ The most common problems with using a PS linkage include the decrease in binding affinity to the target RNA/DNA and nonspecific protein binding. To compensate for this, the PS chemistry is often used together with base and/or sugar modifications which increase target binding affinity.

Phosphorothioamidate morpholino oligomer (PMO) ONs contain a modified backbone in which the phosphodiester linkage and the ribose sugar ring are replaced with synthetic, noncharged morpholine linkages. The advantages to such a chemistry can include high efficacy and specificity, nuclease resistance, aqueous solubility, and low production costs.^66^ However, the incorporation of the PMO chemistry lowers the ON melting temperature, which may be compensated for by increasing the number of bases in the sequence. However, PMOs also have a decreased binding affinity for serum proteins, which leads to rapid blood clearance and limited tissue distribution. The two PMO ONs which have received FDA approval, eteplirsen and golodirsen, suffer from high clearance with 66 and 60%, respectively, of intravenous (IV)-administered ON being recovered in urine within 24 h of administration.^67,68^

**Figure 2:**
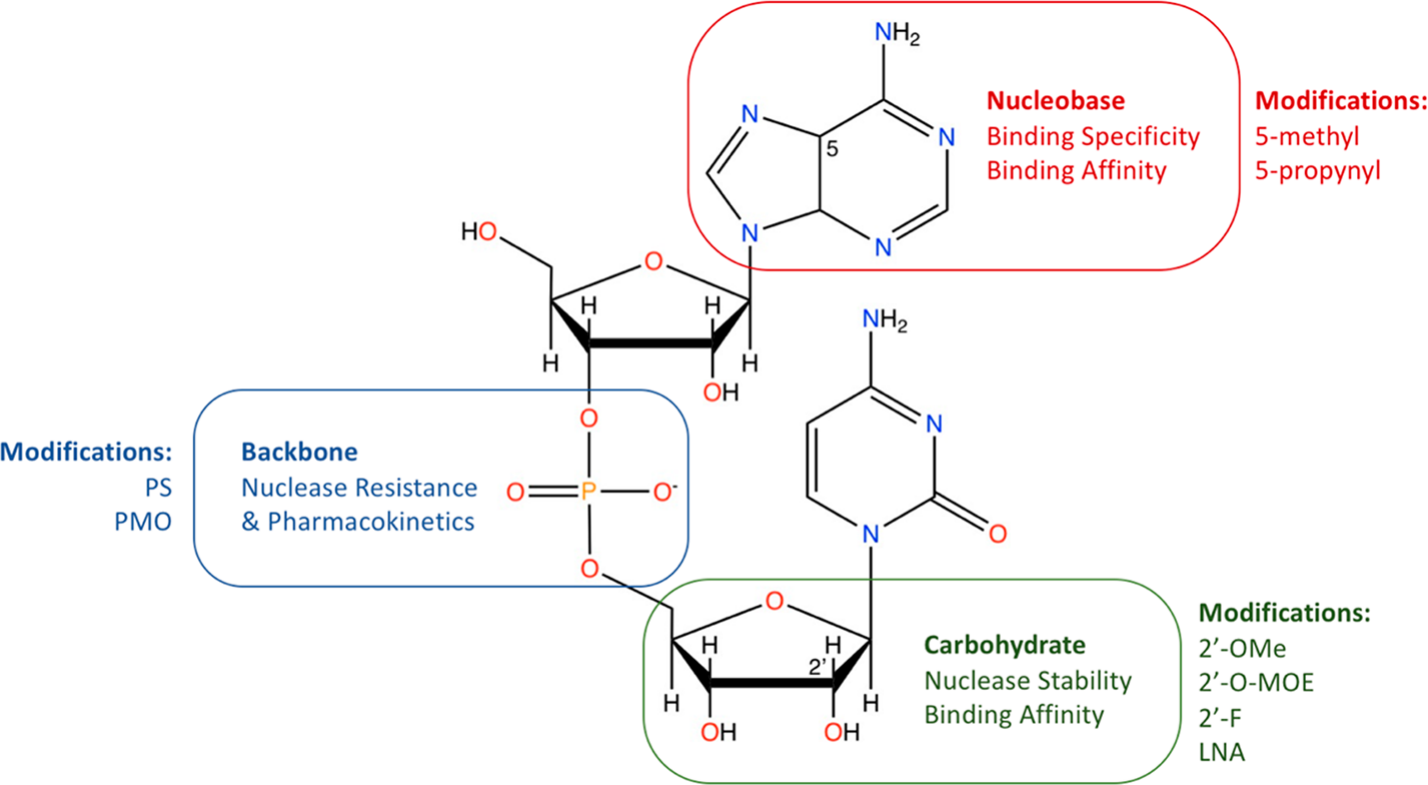
Common chemical modifications for RNA ONs. The three sites for common modifications of RNA ONs include the nucleobase, the phosphate backbone, and the carbohydrate sugar. Advantageous characteristics of modifications are listed for each site, and chemical modifications which are utilized FDA-approved ONs are listed for each. The 5-carbon of the nucleobase and the 2′-carbon of the carbohydrate are annotated with their relevant location number. Abbreviations: PS, phosphorothioate; PMO, phosphorodiamidate morpholino oligomer; 2′-OMe, 2′-O-methyl; 2′-O-MOE, 2′-O-methoxyethyl; 2′-F, 2′-fluoro; LNA, locked nucleic acid. Figure created in BioRender.

## Bioconjugation

Apart from modifying the internal chemical structures on the ON, the possibility exists to chemically modify an ON by conjugating other molecules to it. This can serve the purpose of influencing the targeting and uptake of the ONs on the tissue and cellular level. Additionally, bioconjugates have been shown to alter the kinetics of the therapeutic ONs. Recent work from Alnylam Pharmaceuticals has shown that *N*-acetylgalactosamine (GalNAc), when conjugated to therapeutic ONs, do not actively induce endosomal escape of the ON but rather serve to increase the uptake and storage of the ON within cellular compartments, leading to a sustained therapeutic outcome *in vivo.*(69) Through this approach, a single administration of GalNAc-conjugated siRNA can lead to silencing of the target gene that persists for weeks.

Some of the most commonly utilized bioconjugates include cholesterol and N-GalNAc, but bioconjugates can also include other lipids, sugars, antibodies, and peptides. Cell-penetrating peptides (CPPs) have been used as a bioconjugate, which bolsters the activity of the covalently lined ON. While CPPs have proven effective to increase ON activity *in vitro*, their mechanism of action is not entirely elucidated. It has been shown that CPPs can be designed to work either by pore formation in the plasma membrane or by endosomal destabilization, although the latter is not well-defined.^70,71^

As bioconjugates do not necessarily induce endosomal escape but may rather increase ON activity by other means, bioconjugates lie outside the scope of this review. However, a recent review from Kulkarni **et al.** thoroughly covers the use of this effective delivery strategy in more depth.^72^

## Uptake of Naked ONs (Gymnosis)

Although the hydrophobicity of the cell membrane prevents ONs from permeating freely through, it was shown in 2009 that appropriate dosing can trigger cells to internalize naked LNA ONs in a process known as gymnosis.^73,74^ Therefore, at least one endocytic route for naked ONs must exist. The exact mechanisms driving ON endocytosis are not completely understood. Due to this, ON uptake has been broadly classified as “productive” (yielding a functional outcome in the recipient cells) or “unproductive”.

The uptake process can be divided into three stages: association, internalization, and trafficking, discussed in-depth below. Whether modified ONs are being delivered *via* gymnosis or shuttled into the cell by delivery vehicles, they must escape the endosomal compartment to reach their targets in the cytosol or nucleus. This has proven to be the limiting step in ON delivery, known as “endosomal entrapment”. A considerable portion of recent research has focused on manipulating the association, internalization, and trafficking processes to encourage endosomal compartments to release their cargo. This release, referred to as “endosomal escape”, has become the predominant focus area in the development of RNA therapies.

Additionally, a major factor in determining the therapeutic outcome of an ON lies in its ability to induce the proper immunostimulatory response. Depending on the therapeutic mechanism of the ON, this can include immune evasion or intentional immune recognition and activation. For antisense ONs, it is usually the case that immune avoidance is desired so the ON can reach its target cell without inducing toxicity and other off-target gene effects associated with the inflammatory response.^75^ For example, unmodified ONs can activate the innate immunity by binding pattern-recognition receptors (PRRs) such as Rig-I and PKR, which detect double-stranded RNA in the cytoplasm.^76,77^ In this case, it is desired to design antisense ONs to evade immune recognition. It has been shown that certain chemical modifications such as 2′-OMe-modified uridine and guanidine residues, discussed above, can be incorporated to achieve immune evasion in siRNAs.^78^ Unfortunately, the means by which ONs can induce immune activation are not completely elucidated, and it is not uncommon for ONs which were promising *in vitro* to fall short of their end points *in vivo* due to their immunogenicity. It should be noted that ONs with neutral backbones have not been implicated in immune activation.^79,80^ Conversely, unmethylated CpG-containing ONs activate another PRR, Toll-like receptor 9 (TLR-9), which stimulates the innate immune system. CpG-containing ONs have therefore been tested clinically as vaccine adjuvants and for cancer immunotherapy.^80−82^

### Association

The first stage of uptake, association, occurs as the ON makes contact with proteins on the cell’s surface. PO-ONs show a very low potential for binding to the cell surface. The PS backbone modification has been found to increase binding affinity of naked ONs to proteins on the cell surface. In 1997, it was found that scavenger receptors on endothelial cells were able to bind certain ON species.^83^ More recently, class A scavenger receptors (SCARAs) have been implicated as the principle association target of peptide-conjugated PMOs, tcDNA, and ONs containing 2′-OMe modifications.^84^ Serendipitously, these findings were expanded to include SCARAs as binding targets for PS-ONs when the ONs are administered in high concentrations.^84−86^ Since then, more protein receptors have been identified. These include stabilin-1 and stabilin-2 which were both found to bind PS-ONs with high affinity, inducing clathrin-dependent endocytosis.^87^ Epidermal growth factor receptor (EGFR) has also been shown to directly interact with PS-ONs, cotrafficking them alongside EGF into clathrin-coated pit structures.^88^ Gymnotic uptake is also partly dependent on SIDT2, however to what extent is unclear yet.^89^

As far as we know now, protein binding is the principle association mechanism. It is also important to consider the roles that plasma membrane lipids play in the ON delivery process. In association, lipids coordinate various functions by laterally segregating the membrane proteins into lipid raft structures.^90^ The ability of lipid rafts to form these assemblies of proteins and lipids is critical for internalization to occur.

Other than the PS backbone modification, ON chemistries involving targeting ligand conjugations have been used to increase ON association. Since clathrin-dependent endocytosis has been identified as a productive uptake pathway,^91^ many of these conjugations target membrane proteins known to be internalized during clathrin-dependent endocytosis. These include LDL receptor, transferrin receptor, and certain GPCRs.^83^

Since the 1990s, researchers have also used lipofection techniques to improve the cellular delivery of ONs. This is partially due to the cationic lipids increasing the amount of ON which becomes associated with the cell membrane.^92^ Also, it is also believed that a small percentage of ONs can enter the cell through stimulated macropinocytosis, a less-regulated albeit highly coordinated, triggered pathway of fluid-phase endocytosis.^93,94^

### Internalization

After association, internalization of the ON leads to the entrapment into endosomal vesicles. There are several routes that this could occur by, but all involve two main steps: first, the concentration of materials into a distinct patch on the cell membrane, and subsequently, the protruding or pinching of the membrane-which causes the membrane bud inward, becoming an endosomal vesicle.^95^

Clathrin-mediated endocytosis is the most extensively characterized internalization pathway for productive ON delivery. Clathrin was identified in 1975 as a coat protein comprised of heavy and light chains.^96,97^ Coat proteins are the key players in endocytosis as they induce the formation of specialized membrane patches and sequentially trigger these patches to bud inward.^98^ The clathrin-coated membrane buds are then severed to become endosomal vesicles by dynamin GTPase in a two-step process.^99^ Clathrin-mediated endocytosis is a highly selective process, capable of forming complex vesicles and maintaining precise stoichiometric ratios of the cargo regardless of the vesicle sizes.^100^

Other clathrin-independent internalization mechanisms can also facilitate ON activity. Of these, the most characterized mechanism is caveolae-mediated endocytosis. Caveolin pits are invaginations in the cell membrane which are present in many, but not all, cell types and contain at least one caveolin family protein. They have been implicated in endocytic uptake, as well as maintenance of membrane tension and cell surface area.^101^ These caveolin pits can endocytose a wide range of cargo.^95^ Caveolin pits are dependent on dynamin for vesicle scission similar to clathrin-mediated endocytosis.

Macropinocytosis is another internalization route which does not necessitate the association step. Macropinocytosis involves the ruffling of the membrane to form a protrusion which then collapses, essentially “swallowing” a volume of the extracellular environment. While macropinocytosis is an activated type of endocytosis and therefore requires some cargo association, the internalized volume is large enough for nonselected solute molecules to be internalized from the extracellular environment. Macropinocytosis involves internalization of membrane patches which are much larger than the other endocytic routes (larger than 1 μm).^102^

Several internalization pathways may each contribute to productive ON delivery as all internalization routes converge at the early endosome. In the case of ON delivery, this results in the accumulation of ONs into endosomal compartments regardless of the exact internalization pathway that led them there. This is to the benefit of nuclear ON delivery considering endosomal maturation is a process which generally traffics cargo toward the lysosomes, which are located in proximity to the cell’s nucleus.

### Trafficking

Intracellular trafficking naturally differs between cell types and remains one of the most important processes determining the eventual pharmacological activity of ONs.^103^ Endocytic vesicles typically fuse with early endosomes (EEs) after pinching off from the plasma membrane.^104^ This fusion is mediated by the class C core vacuole/endosome tethering (CORVET) complex which localizes to early endosome membranes.^105^ Early endosomes may then sort their cargo inward to late endosomes (LEs) or multivesicular bodies (MVBs) or recycle cargo back to the cell membrane *via* recycling endosomes for exocytosis.^106,107^ For all cases except exocytosis, the endosomal trafficking will involve a decrease in luminal pH as endosomal compartments mature and eventually converge with lysosomes with an acidic pH between 4 and 5.^108^

The movement of endosomal vesicles through the cytoplasm is a highly regulated process, where vesicles attach to motor proteins in a GTPase-dependent process. In nonpolarized cells, microtubules are arranged radially, enabling endosomal vesicles to move bidirectionally along them toward or away from the nucleus. Kinesin motors are responsible for shuttling organelles inward along microtubules, while dynein motors transport them in the opposite direction.^109^

The Rab protein family, which comprises over 60 GTPases, determines when and how vesicles should move. Rab proteins can recruit a wide variety of effector proteins, making them central to the spatiotemporal regulation of vesicle trafficking by acting as on/off switches for motors, kinases, tethers, and other proteins.^110^ Due to their specific localizations, the Rab proteins are often used in research as markers for various endolysosomal organelles. Rab5 is commonly used as an early endosome marker, while Rab7 is an established late endosome/lysosome marker.^111^

The final step within the trafficking process is the recognition and fusion of vesicles to their targets. The vesicles must be brought within close enough proximity to meet their targets in a process called docking. Docking occurs *via* tether proteins, which are able to recognize markers on the vesicle’s surface to determine whether fusion should occur.^112^ On one end, tether complexes will interact with Rab proteins on the vesicle surface, and on the other end they interact with SNAP receptor proteins (SNAREs).^113^

There are several protein families controlling the fusion process, but at its core, the SNARE complex is responsible for the reaction driving membrane fusion. There are two subsets which form the complex when they meet: t-SNARE proteins located on the target membrane and v-SNARE proteins bound to the vesicle membrane. Formation of the SNARE complex is an extremely energetically favorable reaction, sufficient to overcome the energy barrier to membrane fusion.^114^ It cannot work alone, however, due to a lack of specificity, and there exists a wide assortment of factors and regulators, assuring the vesicles only fuse to their intended targets.^115^

PS-ONs have been shown to quickly progress from EEs to LEs with the help of Annexin A-2 (ANXA2).^104^ ANXA2 colocalizes with PS-ONs in late endosomes, and upregulation of ANXA2 enhances ON activity. ANXA2 reduction caused significant accumulation of ONs in early endosomes and reduced their localization in LEs, ultimately decreasing PS-ON activity.^104^ EGFR, mentioned above involved with association, has also been implicated to assist the endocytic trafficking of PS-ONs to late endosomes, and interestingly, increased levels of EGFR correlates with increased PS-ON activity.^88^

As the endosomal cargo is trafficked along the endosomal system, it will proceed through certain points which have been identified as potential points of endosomal escape.

### Endosomal Escape

The endolysosomal network ultimately have three end points. ONs will either be redirected to the extracellular environment *via* exocytosis, released into the cytosol of the cell where they can reach their targets, or arrested and degraded in lysosomes. In the context of ON delivery, the exocytic and lysosomal end points can be considered as nonproductive. The targets for almost all ONs are generally in the cytosol or the nucleus. The ideal ON delivery pathways would allow the ONs to “hitchhike” along the endosomal pathway toward the nucleus and then escape before reaching the lysosome.

The predominant approaches for encouraging endosomal escape rely on the acidification of endosomal vesicles that occurs as they mature from EE to LE to lysosomes. This acidity is achieved by action of vacuolar-type ATPase (V-ATPase), an ATP-reliant complex which pumps protons into the lysosomal lumen.^116^ The extent of V-ATPase activity may vary between lysosomes, as lysosomal luminal pH and composition can differ depending on their cellular location.^117^ The positioning of lysosomes to the perinuclear region is determined by the endoplasmic reticulum (ER) protein ring finger protein 26 (RNF26) and to a lesser extent by Rab34, which associates to the Golgi apparatus.^118−120^

In the context of ON delivery, this decreasing pH can be buffered with an ionizable or amphipathic delivery agent. One hypothesis, deemed the “proton sponge effect” asserts that buffering the lumen of late endosomes and lysosomes results in an osmotic inflow of water into the endosome, which can lead to engorgement and leakiness due to physical stress on the membrane.^121^

Another approach for enhancing endosomal escape involves exploiting the differences in lipid profiles of the endolysosomal organelles. For example, cholesterol is abundant in the membrane of late endosomes, but it is present at very low levels in the lysosomal membrane.^122^ LDL cholesterol has been observed to enlarge endosomes and increase their volume, which could induce leakage due to mechanical stress.^123^ After LDL has been internalized in a clathrin-dependent manner, it can be released directly from the early endosome, or it can continue to the late endosome where it undergoes hydrolysis and the LDL-derived cholesterol egresses from the late endosome.^124^

Third, the formation of MVBs is another key point for leakage to occur. During the transition from endosome (either early or late) to MVB, inward budding of the endosomal membrane will occur, resulting in the formation of intraluminal vesicles (ILVs).^125^ ILVs, which contain the luminal cargo, can sometimes fuse with the MVB membrane which they are contained within, in a process known as back fusion.^126^ Back fusion is one possible route for ONs to escape into the cytosol and the nucleus.

Lysobisphosphatidic acid (LBPA) has been indicated as an important controller of the fusion cycles of ILVs.^127^ LBPA is necessary for the late endosomal membrane to deform and bud inward, as it does during the formation of ILVs.^128^ LBPA is a phospholipid which is only present in LEs, and has been implicated in controlling endosomal cholesterol levels.^129^ LBPA-mediated intraendosomal trafficking significantly contributes to productive ON release.^130^

**Figure 3:**
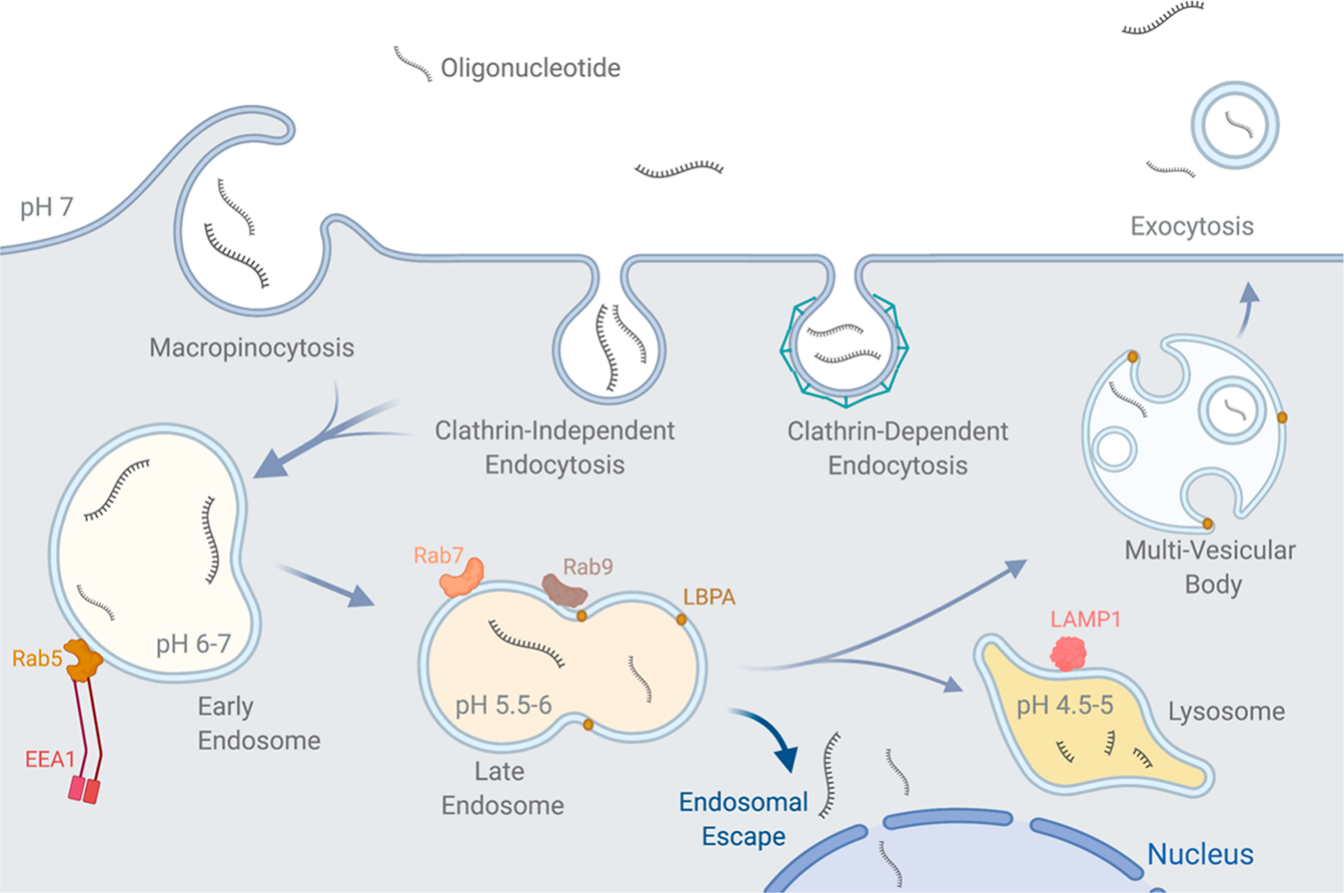
Endocytic uptake and endosomal escape of ONs. The major identified internalization routes of ONs are clathrin-dependent endocytosis, clathrin-independent endocytosis, and macropinocytosis. ON is then trafficked sequentially to the early endosome and sequentially to the late endosome, where it is trafficked to the lysosome or to the multivesicular bodies and exocytosed. Late endosome membrane remodeling and transition to MVB or lysosome have been indicated as likely points of endosomal escape. Commonly used endosomal markers are shown. Abbreviations: Rab, Ras-associated protein; EEA1, early endosome antigen 1; LBPA, lysobisphosphatidic acid; LAMP1, lysosomal-associated membrane protein 1. Figure created in BioRender.

## Nanoparticles for RNA Delivery

In addition to the current repertoire of chemical modifications, there is a growing focus on developing synthetic and biological nanoparticles for ON delivery. In this review, we look into two nanoparticle-based approaches that are used to enhance delivery: lipid nanoparticles (LNPs) and extracellular vesicles (EVs).

## Lipid-Based Nanoparticles for RNA Delivery

Sophisticated synthetic delivery systems are designed for genetic drugs to be used clinically. Lipid-based nanoparticles can be engineered to package diverse cargo for effective therapeutic delivery and are currently the most promising nonviral delivery systems for enabling the clinical potential of genetic drugs.^131−135^ The particle structure of the nanoparticle is dictated by the self-assembled properties of the lipid and cargo mixtures in the specific buffer conditions chosen. Broadly, lipid-based particles can be divided into two key types: liposomes and lipid nanoparticles (LNPs).

Liposomes have a core–shell structure with a uni- or multilamellar lipid bilayer surrounding an aqueous internal core. Many investigated drug delivery systems employ unilamellar lipid vesicles of around 100 nm in size, although depending on the formulation method a significant proportion of multilamellar lipid vesicles may coexist, which can impact cargo loading and delivery.^136^

LNPs lack the internal aqueous core that defines liposomes and instead have a lipid-based core whose structure depends on the lipid and cargo mixtures used.^136^ In some cases, the core is highly ordered and the packing of lipids can be described by specific morphologies such as cubic, hexagonal, micellar or sponge phases. These are typically interspersed with either aqueous pockets or water channels. In other cases, the internal lipid core is less well-defined and is an amorphous structure. These have been extensively characterized in other reviews.^137−139^

The formation of stable LNPs also requires the inclusion of a stabilizing moiety. The most commonly used stabilizers are PEG-based polymers and PEGylated lipids. Different stabilizers and their effects on morphology, uptake, and toxicity have already been summarized previously.^140,141^ Understanding the lipid structures and their response to local environments is key to rational design of LNPs, effective cargo loading and its subsequent delivery.

As described above, the major challenge to implementing RNA-based gene therapies is the delivery to their intracellular targets which is limited by their degradation in biological fluids, and limited tissue targeting and cell penetration.^142^ Here, we briefly describe LNPs as synthetic nonviral delivery systems which have the advantages of being easily designed and manufactured while enhancing the delivery to disease sites and reducing immune system stimulation.

### RNA Encapsulation with Ionizable Cationic Lipids

Efficient loading of diverse cargo into lipid-based therapeutics is complex. Small hydrophobic drug molecules can incorporate into the lipid bilayer while hydrophilic molecules can be encapsulated in the aqueous environment.^143^ For larger cargo, such as proteins and RNA, encapsulation can be less efficient and a direct interaction between the particle and cargo is necessary. Electrostatic interactions, where the cargo and membrane have opposite charges, leads to increased loading *via* a charge association. Cargo encapsulation is performed at low pH where the lipid is protonated and positively charged, while at physiological pH, its charge is neutral and the LNP exhibits near-neutral external membrane surface charge. This is the key particle loading mechanism exploited for LNPs with RNA cargo.^144,145^

In 1987, Felgner **et al.** reported on the formation of complexes between the cationic lipid DOTMA and plasmid DNA, which when formulated with DOPE to make lipoplexes resulted in successful transfection of cells *in vitro.*(146) However, using cationic lipids results in a surface charge on the LNP, and it has subsequently been suggested that this may increase toxicity and lead to rapid surface protein adsorption and clearance by the reticuloendothelial system (RES), as well as undesired side effects.^147−149^

The development of optimized “ionizable lipids” represents one of the most important factors in the clinical success of RNA loaded-LNPs. The term ionizable lipid is typically used in the field to describe amine-containing lipids which are neutral at pH 7 but become positively charged at lower pH *via* protonation of the amine moieties.^150^ These ionizable lipids can be used to efficiently encapsulate negatively charged polymers such as RNA and DNA into LNPs by virtue of charge interactions between the lipids and the ONs during the initial formulation step, which occurs below the lipid p*K*_a_. Many studies have quantified particle formation and functional delivery of ON cargo using ionizable lipids. In some cases, they are the predominant lipid species, and in others, form only a percentage of the total lipid mixture. The self-assembled particle structures formed by the lipid mixtures are driven by the biophysical characteristics of the lipid mixture and their interaction with the cargo. There is some evidence indicating that particle structure may impact cytotoxicity.^151^ However, interpretation of these studies is challenging as particle structure is predominantly tuned *via* composition and it can be complex to decouple differences in toxicity due to composition and structure.

Studies into the chemical structures of ionizable lipids and lipid-like structures (termed lipidoids) that maximize the potency of siRNA delivery have systematically varied the hydrocarbon chain unsaturation, linker moiety, and headgroup.^152,153^ Replacing the DOTMA trimethylammonium headgroup with dimethylammonium yields the ionizable lipid DODMA, with one unsaturated carbon bond per C18 hydrocarbon chain. The level of chain unsaturation seems to be an important parameter. By varying the level of chain unsaturation, Heyes and co-workers reported that the most effective formulations were observed using 1,2-dilinoleyloxy-3-dimethylaminopropane (DLinDMA) which has two carbon–carbon double bonds per alkyl chain, and that more or less unsaturation leads to less effective siRNA gene-silencing *in vitro*. They observed that increased unsaturation led to a decrease in the lamellar (L_α_) to inverse hexagonal (H_II_) phase transition temperature and therefore increased fusogenicity, which facilitates endosomal escape. Notably, however, uptake experiments suggested that despite their lower gene-silencing efficiency, the less fusogenic particles were more readily internalized by cells.^152^

Using DLinDMA as a starting molecule, optimization of the “linker group” between the hydrophobic hydrocarbon chains and the hydrophilic headgroup demonstrated that the introduction of a ketal ring linker resulted in approximately a 2.5-fold increase in potency, with siRNA-LNP formulations using this resulting DLin-KC2-DMA (KC2) lipid exhibiting an ED_50_ of approximately 0.4 mg/kg (*versus* 1 mg/kg for DLinDMA) in non-human primates.^153^ Further variation of the headgroup chemistry, and therefore p*K*_a_ value, showed a tight correlation between lipid p*K*_a_ and the ED_50_ of corresponding LNP formulations. Jayaraman and co-workers observed maximum potency at an optimal ionizable lipid p*K*_a_ range of 6.2–6.5^150^ and identified DLin-(MC3)-DMA (MC3), with a p*K*_a_ of 6.44, as particularly potent in siRNA-LNP formulations for gene-silencing in mouse and non-human primate models.^150^

LNP formulations using this optimized MC3 lipid (see Table 2 for structure) have led to potent delivery vectors, including the FDA-approved siRNA drug patisiran (Onpattro).^154^ Additional applications have been observed by Jyotsana **et al.**, who reported on a highly efficient (nearly 100% uptake) and nontoxic MC3 lipid-based LNP formulation loaded with siRNA targeting the BCR-ABL fusion oncogene found in human chronic myeloid leukemia cells *in vivo.*(155) This study demonstrates that fusion oncogene specific RNAi therapeutics can be exploited against leukemic cells and promises additional treatment options for leukemia patients. MC3 can also be used to safely transfect mRNA in order to express therapeutic proteins. Nabhan **et al.** have demonstrated that RNA transcript therapy can be used for the delivery of therapeutic mRNA to dorsal root ganglia using an MC3-based LNP system.^156^ In rat and monkey models, Sedic **et al.** reported on the safe profile of MC3-based mRNA-loaded LNPs which led to hEPO expression and were well tolerated even above the anticipated efficacious dose levels.^157^ Ionizable lipids developed by Harashima and co-workers use the same DLin tail as MC3 with slight modifications on the amine-containing headgroup, giving p*K*_a_ values of 6.5 (lipid YSK05) and 8 (lipid YSK12-C4).^158^ Formulations including these lipids have shown good *in vivo* delivery of respectively plasmid DNA to the spleen^159^ and siRNA to dendritic cells.^160^ Furthermore, by adjusting the ratio of these lipids in LNP formulations, they reported tuning the LNP membrane p*K*_a_ for targeted delivery of siRNA to liver sinusoidal endothelial cells in mice.^158^

**Table 2:**
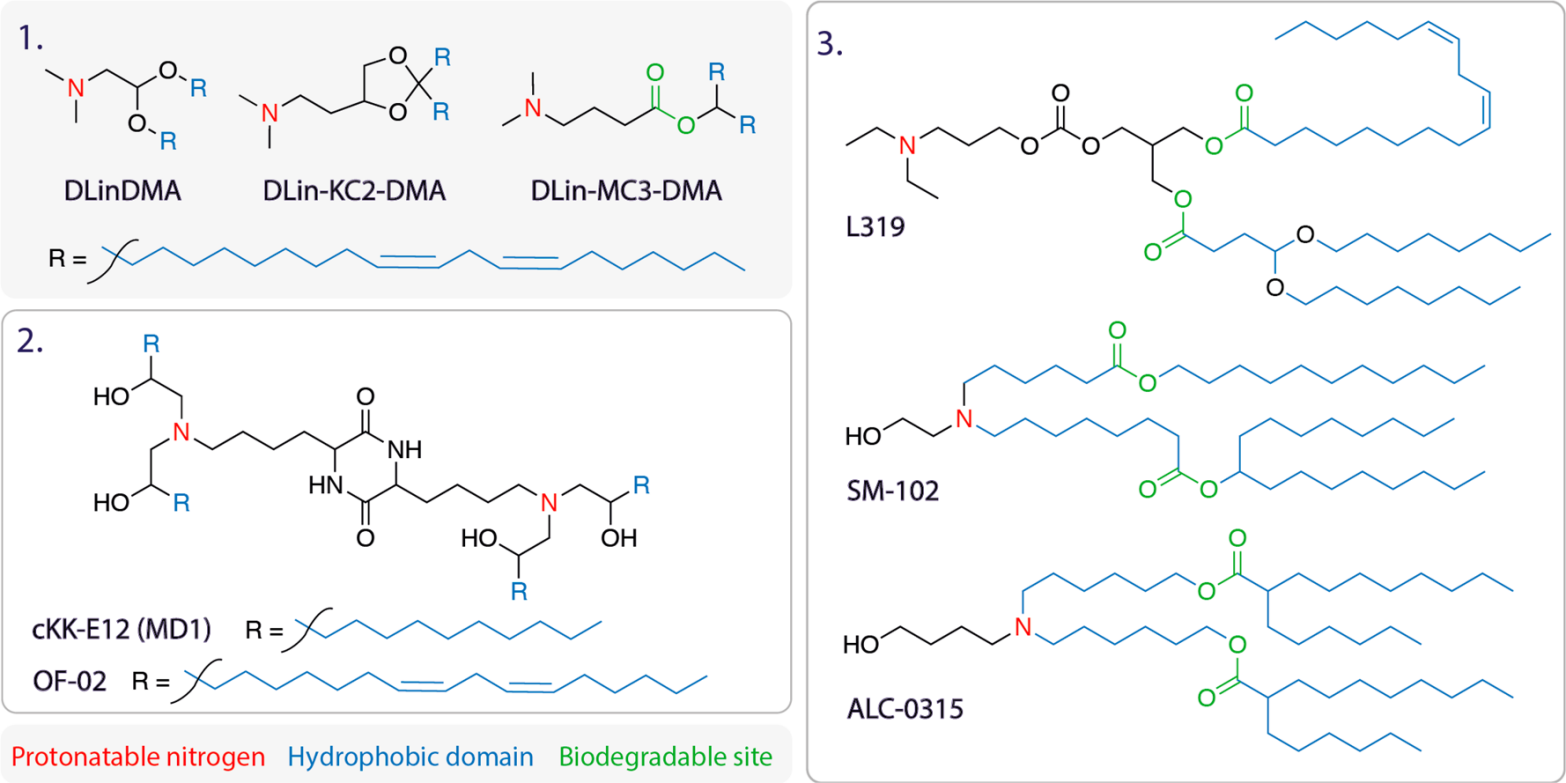
Selected Lipid Structures^a^

a The structures of a few selected commonly used lipids are displayed above. Box 1, ionizable lipid series DLinDMA, DLin-KC2_DMA (KC2), and DLin_MC3-DMA (MC3). Box 2, lipidoids CKK-E12, OF-02. Box 3, next generation biodegradable ionizable lipids L319, SM-102, and ALC-0315. Ionizable cationic lipids are characterized by two functional domains: the ionizable headgroup which contains a protonatable nitrogen (red) and the hydrophobic tail comprising hydrocarbon chains (blue). The structures of lipidoids (examples in box 2) can vary, but generally they also contain protonatable nitrogens and hydrocarbon tails. Next-generation lipids contain an extra functional domain, the site of biodegradable cleavage (green), usually in the form of an ester in the hydrocarbon tail. For LNP formulations using these lipids, see Table 3.

Development of the next-generation of ionizable lipids, which have biodegradable properties such as cleavable linkages that lead to rapid elimination *in vivo*, is already ongoing.^135^ A key motivation has been to improve biocompatibility and tolerability while maintaining high potency *in vivo.*(134) Screening large libraries of lipids and lipidoids synthesized *via* a variety of chemical routes has enabled the roles of structural features in the molecules to be linked to *in vitro*/*in vivo* efficacy. The presence of ester linkages on the lipid tails render these structures biodegradable thanks to esterase activity in the intracellular compartment, and they show enhanced liver clearance *versus* MC3 in non-human primates.^161,162^ Ramaswamy **et al.** also reported successful protein replacement with human recombinant factor IX mRNA in a mouse model of hemophilia B using an LNP formulation that utilized a proprietary ionizable lipid with biodegradable tails, ATX-100.^163^ Here, introduction of ester groups on the lipidic backbone, which can be cleaved by esterases at acidic pH, increased clearance rate with favorable secondary effect outcomes while maintaining potency in comparison to MC3.^134^

Modifications on structures which have showed *in vivo* potency for siRNA delivery include tertiary amino alcohols, where the headgroup alcohol was found to increase activity.^164^ Moderna has in recent years reported the efficacy of aminoethanol headgroup-containing lipids with one linear and one branched alkyl tail, connected *via* biodegradable ester linkers to the tertiary amine, for mRNA therapies.^161,162,165^ One such ionizable lipid structure, SM-102, is a component of Moderna’s COVID-19 vaccine. The Pfizer/BioNTec “Comirnaty” COVID-19 vaccine uses a similar structure, named ALC-0315, with an aminobutanol headgroup.

Anderson and co-workers developed a diketopiperazine-based ionizable lipid, cKK-E12 (also known as MD1), which has been used in LNP formulations for cancer immunotherapy and genome editing.^166−168^ For LNP delivery of mRNA coding for human erythropoietin (EPO), the cKK-E12 formulation potency was superseded by OF-02, which introduced unsaturated fatty chains, thereby increasing mRNA expression compared to cKK-E12.^169^ Further, a biodegradable ester version of OF-02, named OF-Deg-Lin, was shown to promote protein expression selectively in the spleen, whereas the nonbiodegradable OF-02 promoted expression in mouse liver.^170^

In other examples, in formulations utilizing libraries of lipidoids synthesized *via* Michael addition of primary amines and alkyl acrylates and alkyl acrylamides^171^ while alcohol-terminated lipidoids with amide-linked tails improve cell uptake, amine-terminated lipidoids with ester-linked tails impart intracellular delivery by navigating obstacles to delivery further downstream. Notably, in HeLa cells and a mouse model, they observed near complete knockdown of firefly luciferase in siRNA-containing LNPs utilizing mixtures of these two ionizable lipidoids in a synergistic approach, while LNP formulations with the individual lipidoid components were ineffective.^172^ Another variant ionizable lipid named LP-01 (approximate p*K*_a_ 6.1), with an amine headgroup and ester-linked tails, was reported by Finn *et al.* as part of the LNP formulation for the co-delivery of Cas9 mRNA and single guide RNA for transthyretin gene, enabling successful editing of the mouse transthyretin gene in the liver.^173^

In another example, the COATOSOME SS-series comprises two tertiary amines with a range of aliphatic chains, linked by a disulfide bridge. LNP formulations using these ionizable lipids have shown efficient intracellular delivery and low cytotoxicity. The tertiary amine motifs respond to an acidic compartment, such as endosome/lysosome, resulting in membrane destabilization/fusion and RNA cargo release, and the disulfides can be cleaved in the reductive environment of the cytoplasm.^174^ Miao and co-workers reported LNP formulations utilizing lipidoids synthesized from isocyanides in a one-step, three-component reaction. They found that, from a library of over 1000 molecules, lipidoids with cyclic amine headgroups, azole linkers, and unsaturated alkyl tails were the best performing as mRNA vaccines in tumor models *in vivo*, by stimulating adaptive immune cells through the stimulator of interfereon genes (STING) pathway.^175^ In a final example, screening of LNP formulations including lipidoids synthesized by reaction of epoxides with diamines identified the lipidiod C12-200 as having good *in vivo* activity in delivery of siRNA,^176^ mRNA,^177^ and also of self-amplifying RNA (saRNA).^178^ In the latter, the saRNA is complexed to the surface of the LNPs by incubating it with already formulated LNPs, instead of traditionally being incorporated in the formulation mixture, and this was seen to be enough to prevent degradation.

In addition to the structure of the ionizable/cationic lipid, the overall lipid mixture composition, *i.e.*, the ratio of ionizable lipid/cholesterol/phospholipid/stabilizer is crucial for optimized formulations.^179^Table 3 summarizes key structural and formulation optimization studies and details the optimal formulations for specific cargo including the outcome, size/ and formulation method used to produce the LNPs. The majority of studies listed in Table 3 use loaded LNPs prepared with a lipid/nucleic acid charge ratio of 3:1.^180^ It has been suggested that the amine/phosphate charge ratio is a key parameter for cargo delivery, where a critical amount of excess amino lipid is necessary for maximum endosome destabilization. Additionally, PEG-lipid surface coverage and dissociation rate have a significant impact on circulation times.^181^

**Table 3:** Optimized Lipid Nanoparticle Formulations for LNP Delivery of RNA^a^

ionizable lipid(s) (IL)	optimized formulation (mol %)	cargo, size, and method	outcome	ref
DLinDMA	IL (30%)/DSPC (20%)/Chol (48%)/PEG2000-c-DMA (2%)	Luc siRNA 132–182 nm spontaneous vesicle formation by ethanol dilution	80% knockdown in Neuro 2A cells; uptake not rate-limiting for gene-silencing efficiency	Heyes *et al.* 2005^152^
DLin-MC3-DMA	IL (50%)/DSPC (10%)/Chol (38.5%)/PEG2000-DMG (1.5%)	FVII and TTR siRNA 70–90 nm preformed vesicle method	ED_50_ = 0.005 mg/kg (mouse), ED_50_ < 0.03 mg/kg (cynomolgus monkeys)	Jayaraman *et al.* 2012^150^
DLin-KC2-DMA	IL (57.1%)/DPPC (7.1%)/Chol (34.3%)/PEG-2000-C-DMA (1.4%)	FVII and TTR siRNA 64–85 nm preformed vesicle method and stepwise dilution	*in vivo* activity achieved at doses of 0.01 mg/kg in mice (C57BL/6) and 0.1 mg/kg in cynomolgus monkeys	Semple *et al.* 2010^153^
DLin-MC3-DMA	IL (50%)/DSPC (10%)/Chol (38.5%)/DMPE-PEG-2000 (1.5%)	hEPO mRNA 50–130 nm fast mixing precipitation (microfluidic mixing)	LNPs with 1.5 mol % of DMPE-PEG2000 showed highest hEPO production after 20 h; protein expression in hepatocytes ∼6 hEPO molecules/dosed mRNA, adipocytes ∼0.6 hEPO molecules/dosed mRNA	Yanez Arteta *et al.* 2018^180^
DLin-MC3-DMA	IL (50%)/DSPC (10%)/Chol (38.5%)/DMG-PEG2000 (1.5%)	Luc mRNA 83–242 nm microfluidic mixing	albino BALB/c mice (male and female) received subretinal injections of LNPs; LNPs formulated with MC3 and KC2 showed expression that was 2.8- and 3.2-fold higher than DODMA expression; other formulations had significantly lower expression	Patel *et al.* 2019^182^
L319	IL (55%)/DSPC (10%)/Chol (32.5%)/PEG2000-DMG (2.5%)	FVII and TTR mRNA avg. 60 nm spontaneous vesicle formation	ED_50_ < 0.01 mg/kg (mouse, FVII model), in cynomolgus monkeys ∼70% silencing of TTR mRNA relative to control	Maier *et al.* 2013^134^
cKK-E12	IL (15%)/DOPE (26%)/Chol (40.5%)/SLS (16%)/DMPE-PEG2000 (2.5%)	TRP2 and gp100 mRNA 84–108 nm microfluidic chip	C57BL/6 mice optimized LNPs loaded with TRP2 and gp100 slow tumor growth and extend survival in a B16F10 tumor model	Oberli *et al.* 2017^167^
OF-02	IL (35%)/DOPE (16%)/Chol (46.5%)/DMPE-PEG2000 (2.5%)	hEPO and Luc mRNA 75–112 nm microfluidic mixing	female C57BL/6 mice hEPO expression using OF-02 is double that using cKK-E12; biodistribution (firefly luciferase) is similar to that of cKK-E12	Fenton *et al.* 2016^169^

a Various studies aiming to optimize and characterize LNP formulations for mRNA/siRNA treatments are summarized above. Many of these studies involved screening several lipid species, and only the optimized lipid composition is shown here. Additionally, the RNA cargo, nanoparticle sizes, and LNP formation methods are mentioned. The outcomes of the treatment involving the optimized formulations are reported, measured by various means between studies, and briefly summarized.

### RNA-LNP Activity: Structures and Formation Mechanisms

Optimizing production methods for the controlled formulation of LNPs is challenging. From an industrial perspective, samples typically need to exhibit functional and structural reproducibility, long-term stability, scalability, and cost efficiency. Many studies have reported optimization of production methods for laboratory studies to improve the controlled assembly of RNA-loaded LNPs. Particular challenges include control of LNP diameter, encapsulation efficiency morphology, and composition.^145,161,167,181,182^

The required diameter of LNP formulations designed for passive targeting will depend on their clinical application. A 100–200 nm diameter LNP is appropriate to get a reasonable cell uptake. For tumor extravasation and retention, however, a 50–100 nm is more suitable. For targeting the lymphatic system, **e.g.**, bone marrow, 40–50 nm diameter would be a pertinent choice. Considering the size of low-density lipoproteins made in the liver (about 20 nm), for a long-circulating LNP system, a 20–30 nm diameter size will allow access to most locations in the body, except perhaps the brain and muscle. Therefore, control over the particle size is crucial for clinical success.

Tuning the particle size can be achieved using microfluidic formulation and by precisely controlling fluid flow rates, allowing different size distributions to be achieved for identical particle compositions. Changing the scale of formulations may affect their properties, requiring expensive and time-consuming process development. It is possible to form LNPs using numerous techniques including sonication, agitation, homogenization, the spontaneous vesicle formation method, preformed vesicle method, and microfluidic mixing.^131,137,183^ The first three techniques necessitate cargo loading post-LNP formation as the harsh conditions can degrade sensitive cargo such as RNA. With these considerations, the majority of LNPs loaded with ON cargo are currently formulated using the spontaneous vesicle formation method either in bulk or with an automated mixer with two inputs. More recently, the mixing is commonly performed on a microfluidic chip.

In a microfluidic chip, one input channel injects the lipids used in the formulation, typically the ionizable lipid, cholesterol, and some helper lipids such as phospholipids (*e.g.*, DSPC, DOPC) and PEG-lipids which are dissolved in ethanol, and the other channel injects the nucleic acid which is formulated in aqueous buffer at pH 4. The rapid mixing of the solvent and aqueous mediums drives the self-assembly of lipid structures. Recently, many experimental approaches have been employed to optimize siRNA loaded LNPs formulated *via* microfluidics.^184^ In many cases, the resulting LNPs are highly dependent on the formulation conditions used and subsequent dialysis steps. At a pH below its p*K*_a_, the ionizable lipid is positively charged, and therefore, at pH 4, there is an electrostatic interaction between the negatively charged RNA and the lipid structures formed which drives an association between them. At pH 7.4 the ionizable lipid is above its p*K*_a_ and therefore no longer positively charged. After mixing of the lipids and cargo in the microfluidic chip, the solution is subjected to a dialysis to remove the ethanol and increase the pH from 4 to 7. This pH change and the removal of ethanol induces structural changes in the particles and drives the formation of the resulting LNP.^145^ The proposed LNP formation mechanism is vesicle fusion induced by the increased pH during the dialysis step and therefore decrease in the charge on the ionizable lipid. Calculations suggest that for every LNP observed after dialysis at pH 7.4, approximately 36 of the positively charged vesicles formed at pH 4 need to fuse as the pH is increased.^145^ By understanding this process, lipid composition can be revised in order to optimize LNP stability and performance.

Understanding the assembly mechanisms of RNA loaded LNPs is crucial to optimizing formulations. An early mechanistic study used molecular-modeling approaches, cryo-transmission electron microscopy, ^31^P NMR, membrane fusion assays, and density measurements to study mixtures of DLinKC2-DMA/DSPC/Chol/PEG-lipid (40/11.5/47.5/1 mol %). The results suggested that siRNA-LNP systems have a nanostructured core consisting of a periodic arrangement of inverted micelles with aqueous cores, some of which contain siRNA duplexes. The proposed formation involved three key stages; first, rapid mixing between the aqueous siRNA phase and the lipid ethanol phase, second, the association of cationic lipid with siRNA to form hydrophobic nucleating micellar structures, and third, the coating of the nucleating structures by remaining lipids (potentially the PEG-lipids) as they reach their solubility limits in the ethanol/water system.^185^ Further studies have shown that LNP–siRNA systems can exist in a continuum of bilayer and nanostructured micellar structures where the morphology depends on the lipid composition and siRNA content. As the DLin-KC2-DMA content increased beyond 70 mol % siRNA encapsulation efficiencies decreased. This effect was even more pronounced for formulations containing higher percentages of PEG-lipid. Proof of concept data also demonstrated encapsulation of mRNA, plasmid DNA, and gold nanoparticles into LNP systems using microfluidic formulation techniques.^186^

More recent studies of lipid mixtures loaded with siRNA and characterized using cryogenic transmission electron microscopy and small-angle X-ray scattering at clinically relevant siRNA content levels have proposed a revised LNP structure which includes a combination of siRNA-bilayer structures and an amorphous core. Based on structural observations where the size of the amorphous LNP core depends on the amount of ionizable lipid (20–50 mol %), the amorphous core appears to be enriched with the ionizable lipid.^145^ The effect of mRNA on the structure of MC3-based LNPs with encapsulated mRNA has also been reported. In a small-angle scattering study, Lindfors and co-workers observed a disordered inverse hexagonal structure in mRNA-loaded LNPs which was absent in unloaded particles. The also reported localization of the lamellar phase lipid DSPC to particle surfaces^180^ and showed that both size and particle surface structure has a significant effect on intracellular protein production *in vitro*.

Experiments demonstrate that increasing the PEG content up to 5 mol % of the LNP by weight decreases the particle size (27–117 nm), and it has been suggested that the LNP surface is enriched in PEG-lipid.^145,181^ Consideration of the amount and type of PEG-lipid is also crucial for rational design of RNA loaded LNPs. The PEG-lipid is essential to produce a stable LNP population with low polydispersity. However, short chain PEG-lipids are currently preferable to promote shedding of the steric barrier following IV administration to maximize hepatic gene-silencing *in vivo*. Studies using lipid compositions of MC3, distearoylphosphatidylcholine, cholesterol, and PEG-lipid to quantitate hepatic gene-silencing showed increasing the concentration above 1.5 mol % substantially compromises hepatocyte gene knockdown for PEG-lipids with longer chains (C18) but not for shorter chains (C14 and C16). This is attributed to an increased PEG-lipid desorption time *in vivo* for C18 compared to C14.^187^

The amount of PEG-lipid also impacts the LNP size—as mentioned previously, the effect of particle size on activity is crucial for certain applications. By varying the amount of PEG-lipid content, particle size can be controlled, with smaller LNPs being formulated at high ratios of PEG-lipid.^180,181,185^ By altering the PEG-lipid composition, LNP-siRNA particles formulated to have a mean diameter of 78 nm showed maximum FVII gene-silencing *in vivo.*(181)

While the size is controlled by the PEG-lipid content, LNP systems that do not contain enough DSPC to cover an external surface monolayer will incorporate additional cholesterol and/or ionizable lipid in that monolayer, thus disturbing the activity. It was shown that siRNA-LNP systems containing 10 mol % of DSPC exhibit maximum activity for a size of 80 nm, which suggests that to obtain smaller systems with optimized activity, higher levels of DSPC should be incorporated.^181^ This was confirmed in both human adipocytes and hepatocytes, where protein expression levels for 130 nm mRNA-LNP systems differed as much as 50-fold depending on lipid compositions with a constant DLin-MC3-DMA/Chol molar ratio. The results suggest that some of these differences may be attributed to changes in surface composition of the particles and the impact this may have on the ability of LNPs to fuse with the endosomal membrane.^180,181^

The proportions of different lipid species in optimized RNA-LNP systems may vary according to the particular ionizable cationic lipid employed (see Table 3). This expands considerably the possible lipid composition of an LNP system but mostly challenges researchers to find the optimal combination for a particular application. On-going efforts are put on using advanced approaches such as high-throughput screening methods and computer-assisted drug formulation, as well as implementing digitalization and artificial intelligence for developing personalized nanomedicine.

### Biological Interactions: PEG Shedding and the Protein Corona

In biological media, LNP dispersions will interact with the numerous biomolecules present. The exact nature of this interaction and the impact on functional delivery will undoubtedly be complex and systematic studies are needed. It is possible that serum proteins adsorb on to the surface of LNPs which could trigger uptake by surrounding macrophages (*e.g.*, Kuppfer cells) or dendritic cells. The PEG-lipid is crucial to maintain the size and stability of the LNPs before administration and any PEG molecules that are present on the particle surface will also minimize *in vivo* serum protein adsorption. This will facilitate access to tissues other than phagocytes. However, if the PEG-lipid is not optimized for the application and lipid composition it can inhibit cellular uptake. It had been observed that the presence of a long-lived PEG-coating (*i.e.*, PEG molecules with C18 or C20 lipids anchors) can dramatically reduce RNA activity.^181,187,188^ To avoid this, the PEG-lipids have to date mostly been designed to partly dissociate from the LNPs following injection. It is thought that this enables access to the LNP surface and therefore interactions with the biological environment and the target cells. This phenomenon is called “PEG shedding”. NMR studies have shown that the rate of “PEG shedding” is inversely proportional to the lipid hydrocarbon chain length, meaning that formulations with shorter PEG-lipids shed more than those with longer tails.^181,189^ Inhibition of cellular uptake and immune response effects were observed by using a C14 anchor PEG-lipid which sheds off from the surface in few minutes postinjection.^187,190^ The mechanism behind this process and the proteins involved remain obscure; however, it is possible that a synergy between PEG shedding and coating of the RNA-LNPs by biomolecules in the surrounding medium, *i.e.*, coronation, is essential to maintain particle stability, cellular uptake, and functional response.^191^

Coronation, or protein corona formation, is described as the protein adsorption layer that forms and defines the biological “identity” of a particle as well as mediates further interactions between the particles and the biological environment.^192−194^ Protein corona studies provide molecular level insight into mechanisms of cellular recognition, uptake, and intracellular destiny of particles.^195^ Among the different classes of adsorbed biomolecules (*e.g.*, proteins,^196^ lipids,^197^ carbohydrates,^198,199^ and metabolites^200^), the apolipoprotein ApoE, involved in the metabolism of fats in the body, has shown to play a crucial role for LNP uptake in hepatocytes.^181,201^ LNP-siRNA gene-silencing activity was significantly decreased in an ApoE knockout mouse model (ApoE–/−), and activity could be rescued by preincubating the particles with ApoE before administration. In a low-density lipoprotein receptor knockout model (LDLR–/−), LNP-siRNA formulations displayed less gene-silencing activity (leading to higher ED_50_ values) than in wild-type animals. LNP activity could be rescued through addition of a multivalent targeting ligand, *N*-acetylgalactosamine (GalNAc), for the hepatocyte asialoglycoprotein receptor, thereby promoting internalization through an alternative endocytic route.^202,203^ Authors concluded that ApoE association with siRNA-LNP systems plays a major role in triggering LNP uptake into hepatocytes by clathrin-mediated endocytosis *via* the LDL receptor. These observations are in line with a previous study by Gilleron *et al.* reporting that uptake of LNPs *in vitro* occurs *via* macropinocytosis and clathrin-mediated endocytosis.^204^ In this study, 50% reduction of LNP uptake upon downregulation of the clathrin heavy chain-1 was observed and knockdown of the macropinocytosis regulators CTBP1, Rac1, Rabankyrin-5 (but not Cdc42) or the use of EIPA, a pharmacological inhibitor of macropinocytosis, led to a 60% and 70% decrease in LNP uptake in HeLa cells, respectively. Downregulation of caveolin 1 did not modify LNP uptake. In the same cell model but using a different cationic lipid to formulate the LNP, Sahay *et al.* reported that downregulation of Cdc42 and Rac1 led to 80% decrease in LNP uptake, whereas inhibition of clathrin heavy chain-1 and caveolin-1 had little impact on LNP entry.^205^ Together, these studies highlight areas in the design of LNP, which need further optimization and understanding to achieve efficient intracellular delivery.

Since LNP delivery efficiency is influenced by the formation of a protein corona in biological media, one may expect that a healthy individual or a patient suffering of a pathology affecting the serum composition, will have different serum-protein diversity and concentration which could possibly affect the protein-corona composition of LNP and thus alter their activity *in vivo*, as observed for PLGA nanoparticles.^206^ In addition, it is important to note that lipid self-assembly and therefore LNP morphology is driven by biophysical parameters including composition, temperature, pressure, electrostatics and lipid packing which have already been extensively reviewed and are therefore not addressed here.^207−209^ Therefore, the morphology and functional response of an RNA-LNP system administrated into a patient with abnormal body temperature (*e.g.*, fever or low-body-temperature-related syndrome) may differ (*e.g.*, protein-corona formation and delivery efficiency) when compared to a healthy volunteer. To date, LNPs have shown efficient delivery of RNA to the liver using passive cellular targeting (size control and protein adsorption). Compositional variations in LNPs have been demonstrated to enhance LNP accumulation in the lung and spleen;^210^ however, delivery to other organs remains challenging. Like liposomal systems, LNPs can take advantage of “natural” and synthetic targeting processes to actively reach specific tissues and cells. The main strategies rely on proteins, peptides or natural ligands, antibodies or antibody fragments, as well as aptamers. Antibody-mediated targeting has demonstrated success in gene-silencing with LNP systems. As an example, Rameshetti *et al.* used an anti-CD4 monoclonal antibody as a targeting moiety on LNP and showed specific binding, uptake and CD45-silencing in murine CD4 positive T lymphocytes following intravenous administration.^211^ In the study, a dose of 1 mg/kg siRNA, lower than other nontargeted systems to leukocytes, was effective in silencing T-cells in the blood, spleen, bone marrow, and inguinal lymph nodes. It was also demonstrated that two CD4 positive T-cell populations exist, whereby internalization of the targeted LNPs was observed only by the CD4 low-expression level population, leading to 69% CD45 knockdown, while localization of LNPs on the surface of the CD4 high-expression level population did not alter CD45 expression. For B-cell malignancy, Weinstein *et al.* designed an anti-CD38 monoclonal antibody-coated LNP to specifically deliver encapsulated siRNA against cyclin D1 in mantle cell lymphoma cells.^212^ The study showed that treatment induced gene-silencing, suppressed tumor cell growth and prolonged survival of mice. Hyaluronan is a natural ligand of the CD44 receptor, which is often overexpressed on the surface of various cancer cells. Cohen *et al.* have shown that local delivery of hyaluronan-coated LNPs containing siRNA for PLK1 specifically target CD44 cells in a murine glioma model.^213^ The treatment induced internalization of the LNP, robust PLK1-silencing, and cell death of glioma cells prolonging survival of animals. It seems reasonable to wonder whether the protein corona confound the targeting of LNP with specific ligands coated on their surface and if the surface functionality of LNP is preserved in the presence of a protein corona. Nevertheless, it remains possible to control on purpose the corona composition. Zhang *et al.* have provided an elegant example whereby retinol-conjugated polyetherimine nanoparticles selectively recruit retinol binding protein 4 in its corona, enabling targeted delivery of antisense ONs to hepatic stellate cells.^214^

### Intracellular Trafficking and Endosomal Escape

Upon internalization, cargo is sequentially transported through early endosomes, late endosomes, and lysosomes.^215^ The various stages of transport can be evaluated by the time-dependent colocalization with specific markers such as EEA1, as well as Rab5, for early endosomes, and Rab7/9 or LAMP-1 for late endosomes and lysosomes. It is believed that an efficient LNP-RNA transfection relies on an early and narrow endosomal escape window prior to lysosomal sequestration and/or exocytosis. In their study, Gilleron *et al.* have explored the biogenesis and maturation of LNP-containing organelles. Following injection of mice with LNP composed of ionizable lipid, cholesterol, DSPC, and DMG-PEG with gold particles conjugated-siRNA, they estimated that only 1–2% of internalized siRNA was released from moderately acidic compartments sharing early and late endosomal characteristics, which nevertheless lead to a significant knockdown.^204^ Sahay *et al.* have tracked the intracellular transport in HeLa cells of similar LNP system (with different cationic lipid) loaded with siRNA. They estimated that 70% of the siRNA underwent endocytic recycling *via* late endosomes and lysosomes and concluded that efficiency of siRNA delivery by LNP is limited by endocytic recycling.^205^ To block these events and thereby increase opportunities for endosomal escape, Wang *et al.* have inhibited the Niemann Pick type C1, a late endosomal/lysosomal membrane protein involved in endosomal recycling.^216^ The study revealed that the presence of the inhibitor NP3.47 caused 3-fold or higher increases in accumulation of LNP-siRNA in late endosomes/lysosomes and the gene-silencing potency of LNP siRNA was enhanced up to 4-fold in the presence of NP3.47. This is an attractive strategy to enhance the therapeutic efficacy, however, it is believed that a deep understanding of what orchestrates the RNA escape from endosomes will aid the design of safe and efficient LNP systems. It has been shown *ex vivo* that cationic lipids can exhibit the ability to induce nonbilayer structures in lipid systems containing anionic phospholipids.^217^

Biophysical studies where the behaviors of biological particles (*e.g.*, cellular organelles or LNPs) and membrane-membrane interactions are mimicked can provide insights into mechanisms driving LNP functionality. These approaches enable control of the LNP environment (*e.g.*, hydrodynamic flow, controlled pH, protein-corona formation) to track their motion *via* surface-sensitive optical imaging, enabling determination of their diffusion coefficients and flow-induced drifts, from which accurate quantification of both size and emission intensity can be made.^218^ In cells, the two methods that provide robust confirmation of endosomal disruption are transmission electron microscopy and cellular fractionation, but these methods are not amenable to rapid, high-throughput analysis. In contrast, fluorescent microscopy allows to detect endosomal rupture events in living cells with high-content imaging. After the role of Galectin 8, a cytosolically dispersed protein, in innate immunity—in which it functions to detect disrupted endosomes due to high-affinity binding with glycans selectively found on the inner leaflet of endosomal membranes—was discovered,^219^ Wittrup *et al.* used cytosolic galectins (Gal1, Gal3, Gal4, Gal8 and Gal9) to monitor endosomal disruption of LNP in living cells.^220^ They reported that the appearance of Gal8 positive spots temporally coincides with the cytosolic delivery of fluorescently labeled siRNA from LNPs. More recently, Kilchrist *et al.* have established the utility of Gal8 subcellular tracking for the rapid optimization and high-throughput screening of the endosome disruption potency of intracellular delivery technologies.^221^ Galectins are 15 members family of carbohydrates with widespread functions and expressions across cell types. The tracking of endosomal escape events requires the development of live-cell imaging assays which can be used to screen for LNP efficiency on a large diversity of cells. A 30-cell line LNP-mRNA transfection screen identified three cell lines having low, medium, and high transfection that correlated with protein expression when they were analyzed in tumor models. Endocytic profiling of these cell lines identified major differences in endolysosomal morphology and pH, localization, endocytic uptake, trafficking and recycling.^222^ The endocytic profiling and monitoring of endosomal escape events are an important and challenging preclinical evaluation step to support the success of nucleic acid delivery systems and improve their translation to clinical trials.

## Extracellular Vesicles for RNA Delivery

Extracellular vesicles (EVs) consist of a heterogeneous family of nanosized vesicles (overall 40–2000 nm) including exosomes, microvesicles (MVs), and apoptotic bodies. EVs are naturally released by all cells into the extracellular environment and body fluids, playing key roles in different processes including antigen presentation and intercellular communication. EVs are able to transfer molecules from donor cells to recipient cells through the extracellular environment and the bloodstream.^223−226^ The field of using EVs for drug delivery was ignited in 2007 when Valadi *et al.* demonstrated that exosomes from murine mast cells could transfer material to human mast cells, resulting in the presence of exogenous murine protein in the recipient human cells.^227^ Since then, myriad publications have demonstrated the utility of using EVs derived from different sources for delivery of various RNA species.

While LNP-based therapeutics have already reached the clinic, EVs are not far behind. Recent developments in the EV field have led to numerous clinical trials involving EVs as targeted therapeutics.^228,229^ As discussed herein, EVs are complex, multicomponent systems, and therefore, their development as a next-generation drug delivery platform requires expansive elucidation. To this end, the EV field has grown exponentially in recent years. In this section, the engineering and production methods of EVs are outlined with a focus on using EVs to deliver RNA.

### EV Biogenesis

Whether *in vitro* or *in vivo*, cells constantly produce, internalize, and recycle biomolecules and nanoparticulate species including EVs. Several subpopulations of EVs exist and can be classified by various criteria such as their cargo composition, their size and density, or, most commonly, their biogenesis.

The goal of EV biogenesis studies is to characterize how EVs are formed *via* different pathways and how each pathway determines the EV composition and physical characteristics.^230,231^ The two general types of EVs, based on biogenesis, are microvesicles (MVs), which bud from the plasma membrane, and exosomes, which originate from the endocytic pathway. Exosomes are formed through the release of intraluminal vesicles (ILVs) from within multivesicular bodies (MVBs). These can be further classified into smaller subpopulations of EVs based on size, density, and the presence or absence of EV biomarkers. Due to MVs and exosomes being formed in different cellular locations and loaded *via* different packaging machinery, their luminal cargo differs in composition.^231^

The biogenesis of EVs is a heavily discussed subject within the EV research field. Years of research have brought to light numerous, difficult-to-elucidate pathways of EV biogenesis, which are heavily interwoven with other cell functions. For example, exosome biogenesis can be broadly divided into endosomal sorting complex required for transport (ESCRT)-independent or ESCRT-dependent pathways. ESCRT and its associated proteins, such as ALIX, syntenin, syndecan, and TSG-101, have been implicated in ILV formation and exosome biogenesis to varying degrees between cell types. Do to their role in EV biogenesis, these proteins have historically been used as EV biomarkers. However, studies in mammalian cell culture have revealed that a complete disruption of ESCRT function, and therefore the interactions of these proteins, does not abolish ILV formation.^232^ Similarly, ALIX depletion decreases but does not abolish EV production but rather shifts the heterogeneity in protein composition of the produced EVs.^233−235^ These findings imply that numerous pathways for exosome secretion exist with some interdependencies, but exosome production is not completely dependent on any single pathway.

The fact that various EV-packaging machineries exist coincides with the fact that certain EV subpopulations can induce differential effects in recipient cells.^236,237^ Together, these imply that certain subpopulations may be better suited than others for some therapeutic strategies. For example, subpopulations can exhibit differential organ biodistribution profiles *in vivo*.^238,239^ It also suggests that certain subpopulations of EVs may contain more relevant biomarkers than other subpopulations and should therefore be preferentially isolated for analysis in diagnostic settings.^240^ The functional differences between EV subpopulations are not yet fully characterized, and there remains a strong focus in the EV field to better understand EV heterogeneity at the single vesicle level.

Despite the complexity of EV biogenesis, effective approaches to load EVs with specific cargo have been developed. These engineering strategies often utilize the proteins which contribute to endogenous EV biogenesis, such as those mentioned above. In the context of RNA delivery, EV engineering approaches seek to preferentially load RNAs into EVs. This can be accomplished through endogenous of exogenous loading approaches.

### Endogenous Loading of RNA into EVs

Several groups have attempted to map the endogenous RNA profiles of EVs from different species, organs, disease states, and cell types. To date, almost all types of RNA have been found in isolated EVs in both functional and fragmented forms, including miRNA, rRNA (rRNA), long noncoding RNA. The majority of RNA present in EVs is between 20 and 200 nucleotides in size. Several groups have also found that EVs, particularly those of cancer origin, contain full length, functional mRNA.^241−243^ Almost every study mapping the RNA profiles of EVs has revealed that certain RNA species are differentially loaded into EVs. It appears that in some instances, the selection of certain RNAs is due to a specific RNA-sorting machinery, and in other instances this differential loading is simply a biproduct of unspecific, unrelated processes.^244^

It is currently believed that EVs carry approximately half of the total circulating RNA in plasma.^245,246^ This includes coding and noncoding RNA such as miRNA, mRNA, tRNA, and others. Additionally, the different populations of EVs contain distinct RNA profiles, with MVs having an RNA profile most closely resembling the transcriptome of the producer cells while exosomes are enriched in miRNA.^247^ The foremost goal of endogenous RNA loading is to take advantage of the inherent selective enrichment of the desired RNA into EVs. This can be accomplished by either a passive or an active loading process. Passive endogenous loading involves using a construct to overexpress the desired RNA which is then loaded into EVs *via* the cells’ own mechanisms. In this approach, the overexpression vector functions to stoichiometrically increase RNA loading without the need for other vectors which alter RNA loading through molecular interaction. Active endogenous loading, on the other hand, involves the implementation of a recombinant fusion construct which usually contains an RNA-binding domain (RBD) fused to an EV protein.^248^ Active endogenous loading has been used to substantially increase the number of target mRNA loaded into EVs.^249^

Active endogenous loading of mRNA must utilize some RBD which recruits the desired RNA into EVs. Currently, there is a strong focus in the EV field to identify RBDs responsible for the endogenous sorting of specific RNA into the EVs.^250^ So far, these studies have revealed specific RNA-binding proteins (RBPs) such as MVP (major vault protein), YBX1 (Y-box protein 1), and sumoylated hnRNPA2B1 (heterogeneous nuclear ribonucleoprotein A2/B1).^251,252^ The presence of endogenous RBPs implies the existence of protein-binding motifs on the mRNA which is enriched in EVs. Separately, this was confirmed in the identification of a zipcode-like 25 nucleotide sequence in the 3′-untranslated region (3′UTR) of mRNAs enriched in MVs compared to their parental cells.^253^

Active endogenous loading is a well-established technique to load exogenous proteins into EVs. For example, in a screening study comparing several GFP-tagged EV sorting domains, Corso *et al.* found that transient overexpression of CD63-GFP in the EV-producing cells yielded fluorescent EVs which contain 40–60 GFP molecules per vesicle.^254^ Additionally, approaches which use non-human RNA-binding domains exist. For example, Wang *et al.* developed a platform utilizing the HIV-TAT peptide to selectively load mRNA into MVs for functional delivery.^255^ The number of RBDs and EV proteins which are being utilized for active endogenous mRNA loading is continually increasing.

Apart from mRNAs, platforms for loading small RNA species into EVs are being developed. Passive endogenous loading of miRNAs can be achieved by use of a miRNA overexpression construct. For example, it has been shown that HEK293 and COS-7 cells, upon transfection with a plasmid coding for several miRNAs (*e.g.*, miR-16, −21, −143, −146a or −155), release exosomes containing these specific miRNAs. Importantly, these exosomes could deliver the miRNAs into recipient cells, leading to mRNA-silencing.^256^ Similarly, pre-miR-451 has been identified as a pre-miRNA which is highly enriched in extracellular vesicles. As long as the hairpin structure of the pre-miRNA is conserved, the miRNA target sequence can be altered.^257^ By inserting a desired target sequence into the pre-miR-451 hairpin structure, EVs were produced which could functionally deliver the pre-miRNA in an efficacious manner, demonstrating this approach as an effective passive endogenous method of loading functional small RNA into EVs.

One of the biggest experimental challenges with using endogenous EV loading is the inability to prevent carry-over of plasmid DNA, viral RNA, or translated protein into the produced EVs. Overexpressing mRNA is always accompanied by increased protein translation in the EV-producing cells. It is then difficult to discriminate between RNA-mediated effects and protein-mediated effects in the recipient cells. de Jong *et al.* approached this issue with development of a Cas9-based reporter system which relies on EV transfer of sgRNA, enabling measurement of EV RNA transfer on the single-cell level.^258^ While there is a focus within the EV field to address this challenging aspect of endogenous RNA loading, there also exist techniques to load EV cargo in a highly selective manner through exogenous loading.

### Exogenous Loading of RNA into EVs

While the endogenous approaches rely on the production of cargo-loaded EVs, the exogenous approaches focus on loading cargo into EVs once the EVs are already produced and isolated. For some EV cargo which is hydrophobic, this can be achieved simply by co-incubation with EVs. In 2010, Curcumin (an anti-inflammatory therapeutic) was successfully loaded into EVs after co-incubation with isolated EVs at room temperature (22 °C) for 5 min. These EVs were able to protect the curcumin as well as to improve its solubility and functional efficiency *in vivo*, suppressing the inflammation in mouse models.^259^

However, cargo which is hydrophilic must be loaded in a strategic manner. This often occurs *via* harsh physical methods which can compromise EV integrity, decrease immune-compatibility, and induce EV and cargo aggregation or degredation.^260,261^ Electroporation is the most common method to date, in which EVs are electrically stimulated while in a solution of the cargo. The EV membrane spontaneously forms pores in which the cargo can enter the lumen.^262^ This has proven an effective method for loading functional siRNA.^263,264^ In 2018, EVs electroporated with either miR-125b-ASO or Cas9 mRNA + sgRNA were able to functionally deliver their cargo.^265^ When the EVs containing the miR-125b-ASO were delivered *in vitro* to leukemia cells or systemically injected in mouse models, the miR-125b expression was reduced; at the same time, EVs loaded with Cas9 mRNA were transferred, simultaneously with gRNA targeting miR-125b, to leukemia cells (MOML13), miR-125b expression was reduced by 98%.

Another, more recent, exogenous RNA loading technique is to co-incubate isolated EVs with cholesterol-conjugated siRNAs (cc-siRNA). After optimizing the protocol for siRNA loading *via* this method, EVs achieved concentration-dependent silencing of human antigen R (HuR).^266^ Similarly, a hydrophobically modified siRNA (hsiRNA) targeting Huntingtin (HTT) mRNA could be functionally loaded into exosomes *via* co-incubation. Reduction of HTT mRNA was observed after the EVs were injected to mouse primary cortical neurons.^267^ Other methods of exogenous RNA loading include EV transfection, sonication,^268^ extrusion,^269^ and liposome-EV hybrid particle formation.^270,271^

A combination of both endogenous and exogenous methods can also be applied successfully.^272^ For example, targeting proteins can be endogenously incorporated to the EV membrane, and then RNA can be loaded to the isolated EVs *via* exogenous methods. This was demonstrated by Alvarez-Erviti *et al.* by means of endogenously loading Lamp2b-fusion constructs onto EVs and then sequentially loading the EVs with siRNA *via* electroporation.^273^ Similarly, EVs engineered with Lamp2b-Rabies Virus Glycoprotein (RVG) and loaded with siRNA were successfully delivered to mouse brain *via* intravenous injection.^264^ These combination engineering strategies are being continuously optimized and show promise.

**Figure 4:**
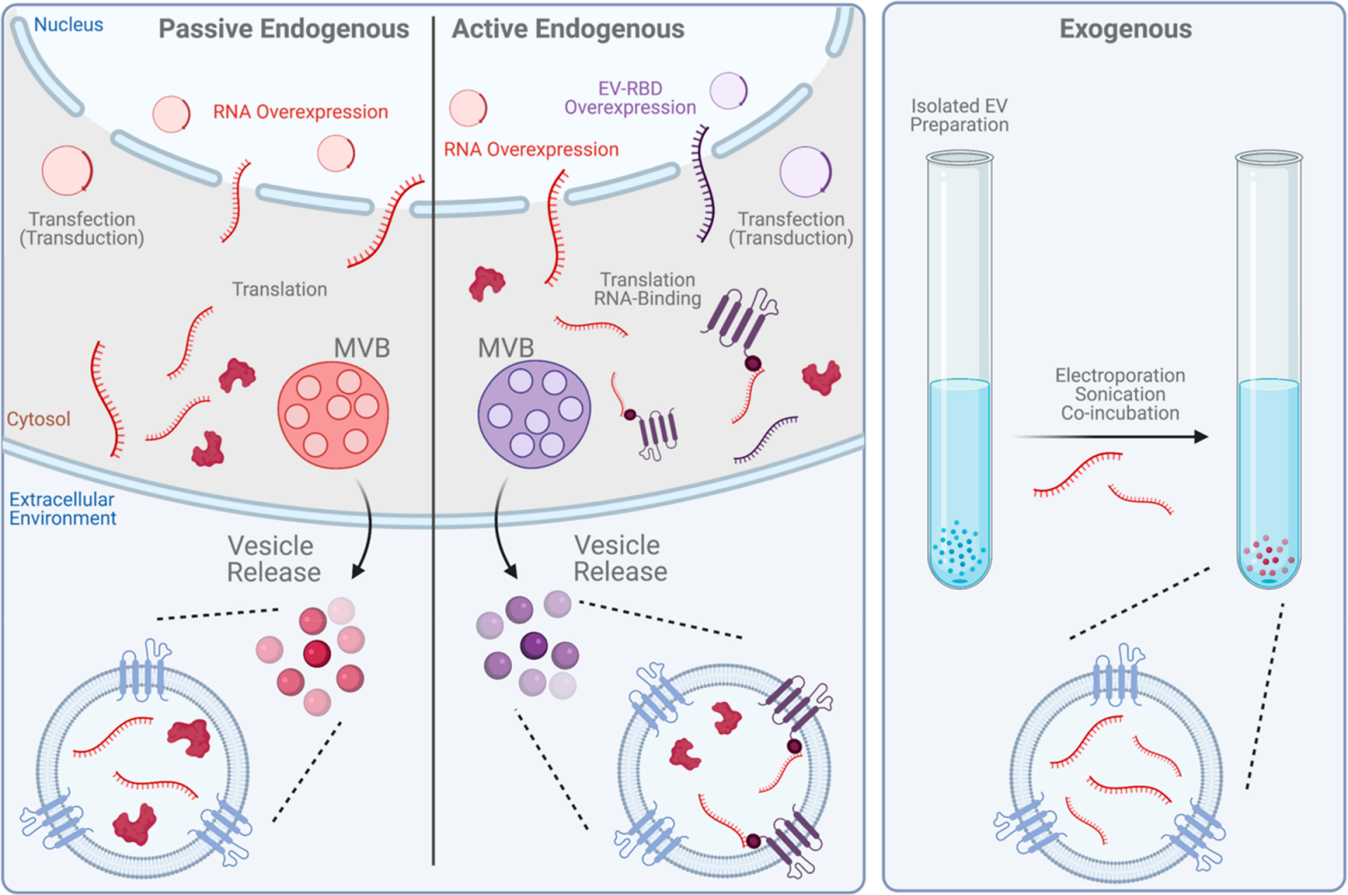
RNA-loading approaches for EVs. EV cargo-loading can be broadly classified as passive endogenous loading, active endogenous loading, or exogenous loading. Endogenous pathways involve transfection or transduction of genetic material into the EV-producing cells. In passive endogenous loading, an RNA overexpression construct leads to stochastic EV loading of an abundantly transcribed RNA. In active endogenous loading, an additional construct comprised of an EV marker protein and an RBD capture the target RNA and shuttle it to EVs during their biogenesis. Exogenous loading occurs after EVs have been isolated and involve physical or chemical techniques to insert RNA into the EV lumen. Abbreviations: RBD, RNA-binding domain; MVB, multivesicular body. Figure created in BioRender.

### EV Fate and Cargo Delivery

The fate of EVs in circulation is believed to be determined by factors such as EV size and the display of surface components which may influence recognition of the EVs by cells. The extents to which EV uptake is determined by EV characteristics or by attributes of the recipient cells remains incompletely characterized, and there are studies suggesting the importance of both. Generally, the mechanisms of EV uptake can be broken down into 3 steps: targeting, internalization, and cargo delivery.

Targeting to the acceptor cell refers to the initial contact and capture of the EV by the acceptor cell. Targeting can occur on a tissue-specific level and a cell-specific level. In regard to tissue targeting, EVs have been shown to have a short half-life in circulation in mice when administered intravenously, with organs showing peak EV internalization at 5 min postinjection.^239^ The organs with the highest EV signal were the liver and spleen.

On the cellular level, several proteins present on both EVs and the surfaces of the acceptor cells have been implicated in EV targeting and capture. These including lectins, proteoglycans, integrins such as ITGB3, and T-cell immunoglobulins.^274−277^ Additionally, exogenous targeting proteins can be utilized to increase or decrease EV targeting to a desired cell type or tissue. As mentioned above, RVG can increase EV targeting to mouse brain.^264^ Conversely, EVs which display CD47 are capable of evading macrophage and monocyte detection, which beneficially increases the EV time in circulation.^278^

The second step, internalization, is also determined both in part by characteristics of the EVs and the acceptor cell types. Early studies of EV internalization identified macropinocytosis as a route of internalization, and since then several other uptake pathways have been identified including receptor-mediated endocytosis and filopodia-recruitment.^279−281^ To date, there are no specific proteins which have been shown completely sufficient and necessary to initiate EV internalization.^282^ However, specific factors have been identified as strongly influencing EV internalization. For one, heparin sulfate proteoglycans (HSPGs) that reside on the acceptor cells are able to bind cancer-derived EVs and the level of HSPG-dependent EV uptake is strongly relevant to the biological activity of the EVs.^277^ In line with this, heparin has been shown to block functional EV transfer between cells.^283^ Additionally, integrins on the surface of EVs from tumors have been implicated as a key component driving the uptake of these EVs.^284^ Together, these findings implicate both positive and negative uptake-regulating factors on the surface of EVs.

The third step, cargo delivery, is dependent on the ability of the EV membrane to fuse with the membrane of the endosomal compartment it is trafficked into. By achieving this fusion, endosomal escape of the EV cargo can be initiated. EV Zeta-potential partially determines membrane destabilization and subsequent membrane fusion. The zeta potential of EVs is influenced by pH and the valency of surrounding ions.^285^ It follows that as internalized EVs are shuttled along the endosomal system, the decreasing pH reduces EV membrane stability, encouraging membrane fusion. The exact endocytic organelles in which this occurs is not fully elucidated.

The lipid composition of the EV membrane and the endosomal membrane are also proposed to influence cargo delivery. Endosomal membranes are constantly undergoing remodeling and recomposition as endosomes mature. The dynamic nature of the endosomal membrane is crucial to EV cargo delivery. Endosomal remodeling is dependent in part on the presence of cholesterol and phosphatidylserine, both of which are present endogenously to EV membranes.^286−289^ The presence of these membrane components may play a crucial role in EV-endosome membrane fusion. Fitting with this, it has been demonstrated that EV cargo delivery is diminished by blocking EV phosphatidylserine.^290^

Many early studies of EV uptake fall short of demonstrating cargo delivery, and instead quantitate only EV internalization. This is usually based on a fluorescent readout which can quantitate uptake events per cell.^291^ However, even if the fluorescent signal is coming from within the cell, it is still unknown if the signal is coming from a functional compartment of the cell such as the cytosol or nucleus, or if the cargo has been arrested in the endosomal system. Recent work has focused on developing approaches to quantitate cytosolic or nuclear delivery of EV cargo. This can be accomplished by means of complementary subunit reporter systems, in which a nonfunctional protein subunit is loaded into EVs and the complementary subunit is expressed in the cytosol of the recipient cell.^292^ This has led to the development of strategies which encourage endosomal escape of EV cargo.

Another recently developed strategy for endosomal escape of EV cargo is to engineer EVs to display fusogenic proteins or peptides on their surface. One of the most promising proteins is the G glycoprotein of the vesicular stomatitis virus glycoprotein (VSVG). VSVG is responsible for membrane attachment and membrane fusion in rhabdoviruses.^293^ In regard to EV engineering, VSVG can be incorporated to the EV membrane through passive endogenous loading.^294^ VSVG can then induce EV membrane fusion in a similar mechanism as it does with viral envelopes, encouraging EV cargo release.^292^ The list of molecular engineering strategies to encourage EV cargo delivery is continuously expanding.

### Current Developments

Even as EVs prove effective in clinical trials, as an emerging next-generation drug delivery platform there are certain areas which remain the focus of ongoing research. For one, EV heterogeneity has been historically addressed on the EV population level. The heterogeneous composition of any EV population adds a layer of complexity to their use. To further resolve this, there is a strong focus on developing single-particle characterization methods for EV analysis.^295^ By examining EVs on the single-particle level, the numerous variables effecting EV activity can be better described.

Additionally, there is a strong focus on elucidating *in vivo* uptake pathways which drive the therapeutic outcome of EVs. As discussed above, there exist quantitative models for investigating EV uptake *in vitro* and *in vivo*, but there is no current consensus regarding the mode of EV uptake *in vivo*.^239,296^ Further, the fate of EV cargo within acceptor cells *in vivo* has yet to be completely elucidated, even though the therapeutic outcomes are quantifiably tangible.

There also exists a practical need in the lack of a standard EV-dosing protocol. As mentioned, EVs are nanoparticles which contain proteins, lipids, nucleic acids, and other biomolecules as cargo. A significant problem with dosing EVs lies in the fact that none of these molecular species correlate perfectly with the overall EV number.^297^ These ratios of the EV cargo to the particle number can be influenced by several factors, including the method of EV isolation and the method of EV quantitation. EVs have historically been quantified by the total particle number, the mass of either protein or lipid, or the presence of specific molecules such as RNA. While it may seem optimal to use the RNA concentration to dose RNA-loaded EVs, the RNA quantitation can be confounded by nonvesicular RNA which is can be present in the final EV preparation in the form of ribonuclearproteins.^298−300^

In conclusion, the potential of using EVs as an RNA therapeutic strategy lies in their ability to combine biological and physical engineering approaches. Each challenge that arises in RNA delivery can be addressed individually and through a range of techniques, as highlighted by the studies discussed herein. As broader genetic engineering approaches develop, the therapeutic EV field will directly benefit.

## Concluding Remarks

The number of clinical and preclinical studies involving RNA therapies, and specifically ON therapies, is rapidly expanding. Only a small number of possible combinations of ON chemistries, targets, and formulations have been investigated to date—a sign that the ON and RNA therapeutic fields are still just in their early days. Nevertheless, ONs have already successfully proven effective to target DNA, RNA, pre-mRNA, and proteins. These qualities firmly establish ONs as a therapeutic class. Separately, biological and synthetic nanocarriers such as EVs and LNPs are each in their own early stages of development but are rapidly gaining attention. As all of these individual advancements come together, the coming years should witness an inflection point in the rate of development of RNA therapeutics.

## Vocabulary

**oligonucleotide**: a short (15–20 bp) strand of natural or synthetic nucleic acids
**endosomal escape**: the point in drug delivery in which the active molecule breaches the endosomal membrane to enter the cell cytosol
**gapmers**: structurally unique ONs which bind to and initiate the degradation of their target RNA in an RNase H-dependent mechanism
**splice-switching ONs**: ONs which act to redirect the splicing repertoire of the target sequence by blocking the normal assembly of the splicing machinery to the pre-mRNA
**extracellular vesicle**: a nanosized lipid-bilayer-bound particle naturally released from cells
**lipid nanoparticle**: spherical vesicles composed at least partially of ionizable lipids
