# Correction to “Delivery of Oligonucleotide Therapeutics: Chemical Modifications, Lipid Nanoparticles, and Extracellular Vesicles”

**DOI:** 10.1021/acsnano.1c09275

**Published:** 2021-10-29

**Authors:** Jeremy P. Bost, Hanna Barriga, Margaret N. Holme, Audrey Gallud, Marco Maugeri, Dhanu Gupta, Taavi Lehto, Hadi Valadi, Elin K. Esbjörner, Molly M. Stevens, Samir El-Andaloussi

In the published article, the structure for L319 shown in Table 2 is incorrect. The corrected Table 2 appears here.

**Table 2:**
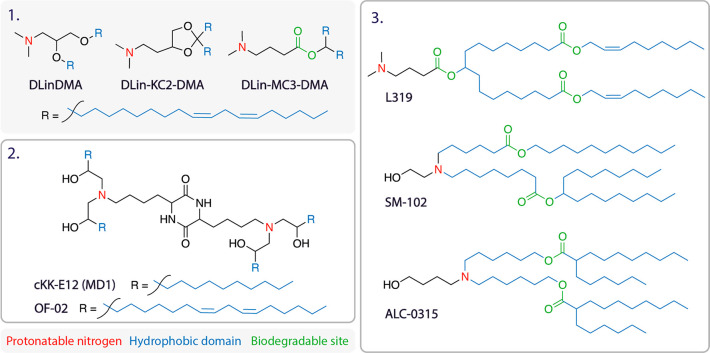
Selected Lipid Structures^a^

a The structures of a few selected commonly used lipids are displayed above. Box 1, ionizable lipid series DLinDMA, DLin-KC2_DMA (KC2), DLin_MC3-DMA (MC3). Box 2, lipidoids CKK-E12, OF-02. Box 3, next-generation biodegradable ionizable lipids L319, SM-102, and ALC-0315. Ionizable cationic lipids are characterized by two functional domains: the ionizable headgroup which contains a protonatable nitrogen (red) and the hydrophobic tail comprising hydrocarbon chains (blue). The structures of lipidoids (examples in box 2) can vary, but generally they also contain protonatable nitrogens and hydrocarbon tails. Next-generation lipids contain an extra functional domain, the site of biodegradable cleavage (green), usually in the form of an ester in the hydrocarbon tail. For LNP formulations using these lipids, see Table 3.

